# Machine learning approaches for electrocatalyst design in water splitting: a review for green hydrogen production

**DOI:** 10.3389/fchem.2026.1894425

**Published:** 2026-07-31

**Authors:** Vamsi Krishna Kudapa, Shoaib Mohd, Vijayakumar Sivasundar, Akanksha Mishra, Santosh Kumar Sahu, Diaa S. Metwally, Mohammed Aman

**Affiliations:** 1 Department of Chemical Engineering, Energy Cluster, UPES, Dehradun, India; 2 Department of Petroleum Engineering, Energy Cluster, UPES, Dehradun, India; 3 Department of Mechanical Engineering, Saveetha School of Engineering, SIMATS, Saveetha University, Chennai, Tamil Nadu, India; 4 Department of Mechanical Engineering, Sharda School of Engineering & Sciences, Sharda University, Greater Noida, India; 5 School of Mechanical Engineering, VIT-AP University, Amaravati, Andhra Pradesh, India; 6 Deanship of Scientific Research, Imam Mohammad Ibn Saud Islamic University (IMSIU), Riyadh, Saudi Arabia; 7 Department of Industrial Engineering, College of Engineering, University of Business and Technology, Jeddah, Saudi Arabia

**Keywords:** electrocatalyst design, green hydrogen, high-throughput screening, machine learning, water splitting

## Abstract

The production of green hydrogen through water splitting requires highly efficient electrocatalysts, but the current trial-and-error-based synthesis or discovery is time-consuming, costly and resource-intensive. Machine learning (ML) provides a powerful, data-driven alternative that can model complex structure-activity relationships across large chemical spaces at orders-of-magnitude speed. This review systematically overviews the life cycle of the electrocatalyst research and development application of ML. First, the thermodynamic and kinetic principles of the hydrogen and oxygen evolution reactions are summarised, along with some well-adopted and accepted activity descriptors. Then we explore data sources, featurization approaches, and algorithms, and discuss the model space, from a simple interpretable model to a graph neural network to a generative model, in the context of the ML toolkit. Strategic applications are discussed for high-throughput virtual screening of alloys and single-atom catalysts, as well as multifunctional activity prediction for overall water splitting, and stability optimisation under operating conditions. The topic of emerging frontiers is highlighted, including high-entropy alloys, amorphous materials, and linking atomic-scale understanding to device-level performance through integration with density functional theory. Finally, the problems of data scarcity, model interpretability and the discrepancy between computational predictions and industrial implementation are discussed, along with future directions for closed-loop discovery and self-driving laboratories. Incorporating ML into electrocatalyst design and combining it with autonomous experimentation will revolutionise this process, from simulation to energy solution, dramatically speeding it up.

## 1 Introduction

Given the increasing worldwide energy demand and the imperative to decarbonize industrial economies, interest in sustainable energy carriers has surged (Li et al., 2023). Hydrogen has become an interesting candidate since it is a zero-emission fuel of the high gravimetric energy density category and can be produced from renewable-power-driven pathways (Gao et al., 2024). Among different production pathways, an electrochemical route supplying hydrogen through water splitting is dominant: it is the most efficient and sustainable pathway for high-purity hydrogen production when coupled with renewable electricity (Li et al., 2023). However, these technologies have not been well-promoted for the moment owing to two barriers: their high energy consumption for the slow oxygen evolution reaction (OER) and the cost of using efficient electrocatalytic (Gao et al., 2024). At the anode, the oxygen evolution reaction has terrible kinetics due to multi-electron transfer paths and the lability of a few other oxygenated intermediates. At the cathode, while more favourable in a kinetic sense, hydrogen evolution still requires catalytic surfaces that bind intermediates optimally per the Sabatier principle. These half-reactions, in total, require electrocatalysts capable of lowering the activation barriers, along with being characterized by rapid charge transfer kinetics and stability owing to the harsh, corrosive operational environment. Traditional catalyst discovery, rooted in iterative trial-and-error experimentation, has thus far been inherently incapable of satisfying these demands. This Edisonian exploration is slow, labor intensive and expensive in terms of resources; it can take months or years at this scale basis to simply peck and poke at how small chunks of the composition landscape are (Ding L. et al., 2024; Bushion et al., 2026; Li et al., 2023). However, the majority of possible alloys, oxides and single atom catalysts remain uncharted, and there are not many clear pathways to pursue. The whole concept of ML is a change of paradigm in itself, and moves the domain of knowledge discovery. The discovery of two-dimensional semimetals with atomic precision in just orders of magnitude lower in cost, with calculations at near DFT level of accuracy, is made possible by data-driven (ML) statistical models to screen thousands of candidates *in silico* and calculate adsorption energies at near (DFT) level of accuracy (Ding L. et al., 2024; Wang C. et al., 2025). In our example, this can involve modelling subtle dependencies that are not intuitive, such as including crystal structure, electronic configuration, and the conditions used to synthesize the material, properties of which are described in high-dimensional vectors.

The effect is not merely straightforward acceleration. Machine learning unlocks compositional spaces previously considered unreachable: high-entropy alloys, where an abnormally large number of elements must coexist with one another; amorphous materials with no long-range order; and multi-principal element systems that are not amenable to classical scaling relations (Tran et al., 2023). In many existing ML models developed with large data sets (both high-throughput and empirical), they have been coupled and used to predict things like which catalysts resist galvanic corrosion when in chloride environments or handle anodic competition during seawater electrolysis, enabling the optimization of stable electrocatalysts for in-situ use within oceans due to their driving force (Din et al., 2025; Sead et al., 2026). Combined with quantum mechanical calculations, ML potentials enable simulations of thousands-of-atom interfaces to help clarify the synergistic role of solvent, surface composition, and morphology in modulating catalytic activity (Keith et al., 2021). Herein, we present a broad overview of the machine-learning toolbox for electrocatalyst design in water splitting. We begin with electrochemical basics—the thermodynamics and kinetics that govern HER and OER, key performance metrics, and the scaling relationships that define common catalyst design. We then turn to the data ecosystem that underpins ML applications: sources and curation strategies, featurization methods that convert atomic structures into machine-readable representations, and an algorithmic landscape spanning interpretable regression models to state-of-the-art graph neural networks. While there have been a number of recent reviews on machine learning for electrocatalysis, most have focused on the development of a specific algorithm or a class of catalysts. This review takes a holistic approach to the catalyst life cycle, integrating fundamental concepts in electrochemistry, catalyst descriptors, materials databases, feature engineering, machine learning algorithms, explainable artificial intelligence, generative AI, autonomous experimentation, and future self-driving laboratories into a single framework. Moreover, in this review, the current methodologies are critically examined, unresolved challenges (such as dataset bias, model uncertainties, interpretability, and the prediction of long-term catalyst stability) are highlighted, and future research directions towards fully autonomous catalyst discovery platforms are suggested.

## 2 Electrochemical foundations and catalyst performance descriptors

### 2.1 Thermodynamics and kinetics of the hydrogen and oxygen evolution reactions

Electrochemical water splitting is the electrochemical decomposition of liquid water into gaseous hydrogen and oxygen through a pair of thermodynamically coupled half-reactions: the H_2_ evolution reaction (HER) at the cathode and the O_2_ evolution reaction (OER) at the anode. A mechanistic perspective of the thermodynamic limitations and kinetic challenges that shape these reactions is crucial to building a foundation for rational catalyst design.

**FIGURE 1:**
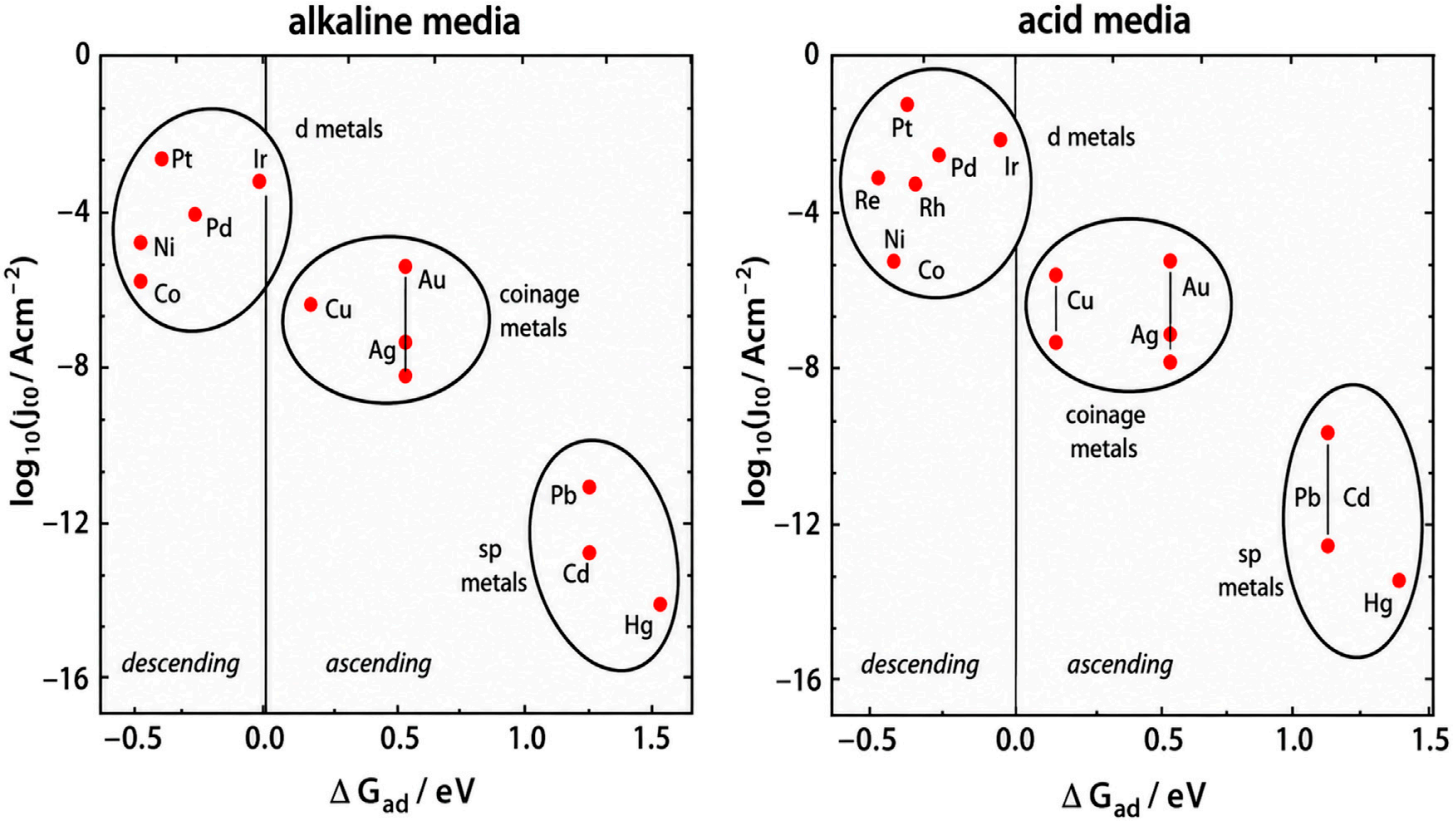
Volcano plots for the hydrogen evolution reaction (HER) in acid (right side) and alkaline (left side) aqueous solutions, showing exchange current density (j_0_) as a function of hydrogen adsorption free energy (ΔG_ad_). Data points represent densely-packed metal surfaces (mostly fcc (111)), with error bars indicating experimental variations. Oxide-covered metals are excluded. The ascending branch shows increased rate with more favorable (negative) ΔG_ad_, but no clear descending branch is observed except for Ni and Co. (Quaino et al., 2014).

The hydrogen evolution reaction (HER) is an electron-hopping multi-step process and can be carried out by the Volmer Heyrovsky or the Volmer–Tafel mechanism in an acid medium (Gao et al., 2024; Srinivasan et al., 2026). This response begins by proton adsorption on the catalyst surface (Volmer step) and derives an adsorbed hydrogen species. This intermediate is then desorbed in an electrochemical recombination reaction with a second proton and electron (Heyrovsky step) or in a chemical recombination reaction between two adjacent adsorbed hydrogen adatoms (Tafel step). In alkaline media, the mechanism changes again: rather than protons, water molecules dissociatively adsorb, supplying hydrogen atoms and adding yet another kinetic complication. The hydrogen adsorption Gibbs free energy, ΔGH, is the most important thermodynamic descriptor of any electrode material: an ideal catalyst binds hydrogen neither too strongly (poisons the surface) nor weakly (prevents adsorption), ΔGH ≈ 0 eV. As noted above, this relationship is often depicted in the HER volcano plot, in which exchange current density is plotted as a function of hydrogen adsorption free energy (ΔGH), thereby illustrating the Sabatier principle in electrocatalysis, as shown in Figure 1.

Figure 1 represents volcano plots of the hydrogen evolution reaction (HER) in acid (a) and alkaline aqueous solution (b), where j_0_ is plotted as a function of ΔG_ad for bulk transition metals with densely-packed metal surfaces (mostly fcc(111)). The error bars are the average of experimental variations available in the literature; oxide-covered metals were omitted, owing to a potential disregard for the descending branch of data. The upward trend of the branch clearly indicates that as ΔG_ad approaches more negative values (more favorable), catalytic activity continues to increase, in agreement with the Sabatier principle. On all d-band metals but Ni and Co, a clear descending branch is missing (indicating that Sabatier’s principle adjuncted with the second requisite of catalytic activity does not adequately characterize HER kinetics). This suggests that extra descriptors like d-band position, metal–hydrogen orbital overlap are needed for rationalizing electrocatalytic activity.

Metals (Pt-group) are notably clustering in the apex of the crater, whereas mild attractions of MoS_2_ lead to continuous investigations on HER catalysts based on non-precious metals. The kinetic penalties in the oxygen evolution reaction are far more pronounced. Such a four-electron, four-proton transfer happens through a succession of adsorbed oxygenate intermediates (OH, O, and OOH), each requiring different stabilization energies (Hu et al., 2024). In alkaline media, the standard reaction pathway is OH^−^ → OH + e^−^; OH + OH^−^ → O + H_2_O + e^−^; O + OH^−^ → OOH + e^−^; and OOH + OH^−^ → O_2_ + H_2_O + e^−^. First, for acidic electrolytes, water serves as the oxygen source, resulting in inverted intermediate energetics. In fact, the overpotential required to kinetically inject into OER, typically 300–400 mV on the best catalysts, is simply a measure of these energy losses added up across each respective sequential elemental reaction step. This slow kinetics is the key reason for the decrease in overall energy conversion efficiency in water electrolysis. Thus, OER emerges as a performance-limiting step (Seh et al., 2017).

### 2.2 Key performance metrics: from overpotential to durability

More generally, assessing electrocatalyst activity requires a battery of quantitative metrics spanning intrinsic activity and pragmatic viability. Overpotential, η, is the additional voltage applied beyond the thermodynamic equilibrium potential of a reaction required to drive it at some specific current density. At a typical current density for various catalyst activity comparisons (10 mA cm^−2^), state-of-the-art platinum faces virtually no overpotential for HER (η ≈ 20 mV), and iridium oxide has η ∼300–400 mV typically for OER (Li, 2022). The Tafel slope, which is in the linear portion of a plot of overpotential versus log current density, provides additional information about reaction kinetics and the rate-determining step; for example, slopes around 30 mV dec^−1^, 40 mV dec^−1^, and 120 mV dec^−1^ imply that the process is Tafel-, Heyrovsky-, or Volmer-limited.

**FIGURE 2:**
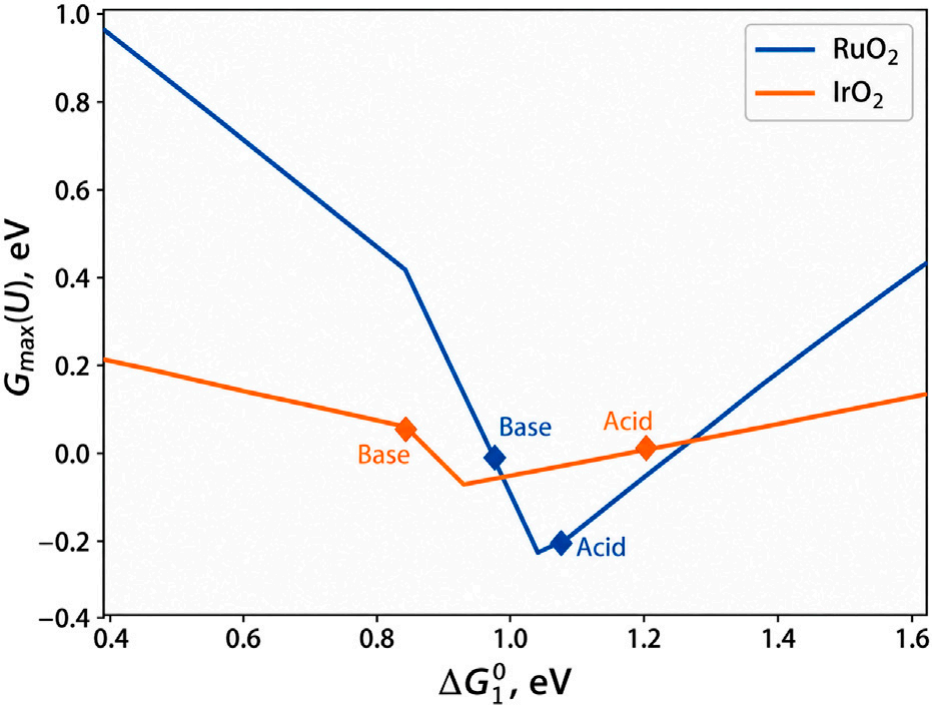
Generalized OER volcano plots comparing RuO_2_(110) and IrO_2_(110) at U = 1.60 V vs. RHE, demonstrating the influence of the *O vs. *OH scaling relation intercept on catalytic activity. The volcano apex shifts based on the scaling parameters, with RuO_2_ showing higher intrinsic activity than IrO_2_ under both acidic and alkaline conditions—consistent with experimental observations. This illustrates the thermodynamic limitations imposed by scaling relations on OER electrocatalysts (Sokolov and Exner, 2024).

Exchange current density, j_0_, is a metric for the intrinsic rate of charge transfer at equilibrium, which can be thought of as a material-specific, overpotential-independent activity descriptor. Figure 2 illustrates how the original performance metrics are translated into actual catalyst designs by employing multiple materials engineering methodologies to simultaneously optimise the number of active sites and intrinsic activity, which, in turn, are connected in series to improve overall catalytic activity.

Figure 2 presents Generalized volcano plots for the oxygen evolution reaction (OER) of RuO_2_(110) and IrO_2_(110). Volcanos are generated by first comparing activity under conditions where 1.60 V vs. RHE is applied), then selecting that value to plot all surface combinations. The thermodynamic constraints of scaling relations between the O and *OH reaction intermediates are shown through the volcano relationship. The intercept (ξ′_1_) of the *O vs. *OH scaling relation is critical in determining both volcanic position and shape, with values ranging between classes of materials. In both pH conditions, RuO_2_ always shows more intrinsic activity than with respect to IrO_2_ which is in agreement with experiments. Moreover, the experimentally derived adsorption free energies (ΔG_1_^0^) for both catalysts are near the volcano plot apex—it is presumed that such bonds of *OH are near optimal. This figure illustrates that scaling relations can restrict maximum OER activity, whose optimization is possible by tuning the *O vs. *OH scaling intercept—not the often emphasizedOOH vs. *OH relation—provides a guideline for better OER electrocatalysts discovery.

These strategies highlight that catalyst design is a balance between the approach of maximizing available active sites and optimizing their intrinsic catalytic properties to achieve improved activity. Unsurprisingly, HER platinum demonstrates a j_0_ ≈ 1 mA cm^−2^, on the order of magnitude higher than its non-precious-metal counterpart. The lack of selectivity toward undesired products is assessed using faradaic efficiency (FE) measurements, which are particularly critical for the OER, as carbon corrosion and chlorine evolution pathways that compete with oxygen formation during seawater electrolysis can divert electrons from the targeted pathway (Chen et al., 2025). Prolonged stability determines functionality despite governing considerably after early action. Accelerated failure procedures almost always consist of thousands of galvanostatic cycles or 100 s of hours of device operation, distilling mechanistic causes that include changes in active-site dissolution, surface reconstructions, particle agglomeration, and corrosion of the catalyst support (Grim et al., 2020). Recently, high-entropy ruthenium oxide catalysts were demonstrated to achieve 1500 h of stability at 100 mA cm^−2^ with extremely low degradation (Qian et al., 2025), thereby offering a promising alternative to the long-standing shortcoming of conventional OER electrocatalysts.

### 2.3 Primer on major electrolyzer technologies and their catalyst demands

Different electrolyser architectures can lead to distinct operational environments, each requiring specific catalyst properties. Proton exchange membrane electrolyzers can also operate in very acidic conditions (pH ≈ 0–2), featuring rapid start-up, high current densities (1–4 A cm^−2^), and small system footprints. This acidic and corrosive environment, along with large anodic overpotential, leads to a very small window of viable OER catalysts, which are mainly precious metal-based oxide (primarily ruthenium and iridium), resistant to dissolution at the aggressive conditions (Wang T. et al., 2022). Overcoming this limitation, however, necessitates catalysts that can mediate potential-free reaction with sub-ppm amassment of active sites. The prohibitive cost and meagre worldwide supply of iridium (annual production < 9 tons) render a major bottleneck for gigawatt-scale PEM implementations, while redirecting considerable research towards both iridium-free substitutes and ultra-low-loading architectures (Pham et al., 2021).

The alkaline electrolysers are operated in a concentrated KOH electrolyte (pH ≈ 14), providing a less corrosive environment that enables earth-abundant transition-metal catalysts. The nickel, cobalt, and iron-based materials are the most investigated systems in this regard and show great OER activity in alkaline media, with that of NiFe layered double hydroxide reaching close to the precious metal benchmark (Luo et al., 2022). Disadvantages of alkaline systems include carbonate precipitation from ambient CO_2_, reduced current densities, and hydrogen crossover, which requires thicker membranes and larger system footprints.

Anion exchange membrane electrolyzers combine the strengths of both architectures: they operate under alkaline conditions suitable for non-precious catalysts while retaining the size and fast response characteristics of membrane-based systems. Among these, both the oriented AEMs and polyelectrolytes that meet durability, ionic conductivity, and stability requirements at high current densities still constitute one of several dynamic research frontiers (Wu et al., 2025). Direct seawater electrolysis is a particularly promising pathway that utilises these large marine resources increasingly effectively; moreover, the catalysts must also resist chloride corrosion-fouling due to precipitation and side reactions competing for chlorine evolution (Din et al., 2025).

Comparison of the key water electrolysis technologies side by side: PEM, alkaline, AEM and direct seawater. Table 1 summarises the operating conditions (pH and current density), the major benefits, the need for catalysts, typical materials, and the major development challenges for each. It provides a link between the previous section on electrochemical kinetics (HER/OER) and the device’s realistic requirements and identifies the changes in catalyst use needed due to the presence of acid, alkali, or chloride. This table further serves the purpose of the later sections by listing the stability required and cost restrictions desired by the ML.

**TABLE 1:** Comparison of electrolyzer technologies and their catalyst demands (Din et al., 2025; Pham et al., 2021; Wang T. et al., 2022).

Electrolyzer type	Operating conditions	Key advantages	Catalyst requirements	Typical catalysts	Challenges
Proton Exchange Membrane (PEM)	Acidic (pH 0–2), 1–4 A cm^−2^	High current density, fast response, compact design	Acid-stable, high OER activity, low metal dissolution	IrO_2_, RuO_2_, Pt/C	High cost, scarce Ir/Ru, limited durability
Alkaline (AEM)	Alkaline (pH ≈ 14), 0.2–1 A cm^−2^	Non-precious metal catalysts are less corrosive	Alkaline-stable, high conductivity, carbonate resistance	NiFe-LDH, Co_3_O_4_, NiMo alloys	Carbonate precipitation, lower current density
Anion Exchange Membrane (AEM)	Alkaline (pH 12–14), 1–2 A cm^−2^	Non-precious metals, compact design, fast response	Alkaline stability, membrane durability, high ionic conductivity	NiFe-based, Co-based, perovskite oxides	Membrane stability, limited long-term durability
Direct Seawater	Near-neutral to alkaline, Cl^−^-rich environment	Abundant water source, no freshwater demand	Chlorine corrosion resistance, high OER selectivity	NiFe-LDH, high-entropy alloys, MnO_2_	Chloride oxidation, fouling, precipitation

### 2.4 Established activity descriptors and scaling relations

**FIGURE 3:**
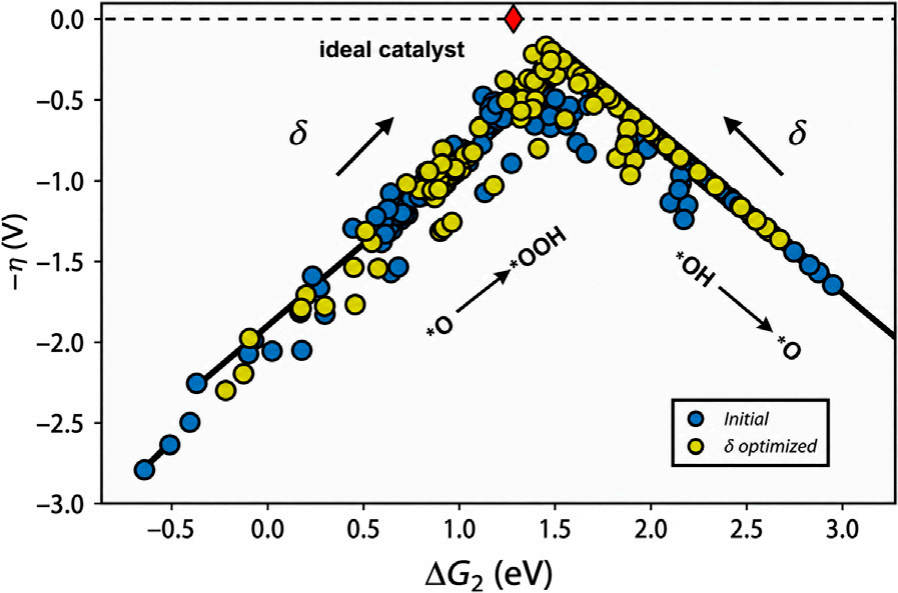
Volcano-type relationship illustrating the dependence of OER overpotential on intermediate adsorption energies, highlighting the limitation imposed by scaling relations and the concept of an ideal catalyst (Govindarajan et al., 2019).

The Sabatier principle can be simply applied to the design of catalysts; too strong or too weak an intermediate interaction will not give the most active catalyst. Platinum is on top of this busy volcano plot which is now dominated by ΔGH for HER as the main descriptor of hydrogen adsorption free energy, HER. For OER, the situation is even more complicated as the energies of binding with the several intermediates (OH, O, OOH) are also coupled to some extent by the interactions; thus, it is not possible to tune the others binding energies independently (Kibsgaard and Chorkendorff, 2019). This mutual dependence is presented as “scaling relations” – linear relations between the adsorption energy for other intermediates of the class of catalysts. This leads to ΔGOOH of about 3.2 ± 0.2 eV, which is equal to ΔGOH for OER, and corresponds to the same bonding configurations found at the top of metal oxide surfaces. This scaling principle suggests a thermodynamic limitation: maximizing one intermediary will always come at the price of optimizing another intermediary, and the optimum overpotential is always the very minimum (0.2–0.3 eV), which is not attainable in standard means of surface engineering. The same very fundamental scaling restriction can be expressed for OER activity as volcano curve (Figure 3) that implies that as the deviation from optimal mid-point binding energetics increases, so does the intrinsic limiting minimum achievable OER overpotential (Li, 2022).

Figure 3 is an estimate of how far intermediate binding energies can deviate from scale, thus motivating new design strategies that go beyond existing scaling relationships. Avoiding these scaling relations is a major challenge in the design of next-generation catalysts. These approaches include stabilization of intermediates by second coordination sphere effects, utilizing ensemble effects on multimetallic surfaces, and active site engineering with respect to conventional adsorption geometries (Govindarajan et al., 2019). Specific classes of materials, such as high-entropy alloys (HEAs), offer exciting opportunities: the multitude of local coordination environments they present fosters a continuous spectrum of possible adsorption sites that can potentially facilitate independent adjustment of multiple intermediates (Xu S. et al., 2024). Similarly, metal–support interactions and coordination-environment engineering help single-atom catalysts attain uncommon binding configurations (Tong et al., 2022). Found only at deeper scales, these well-known descriptors and their associated scaling relations form the theoretical foundation on which machine-learning models can base themselves to further develop and ultimately surpass. With training on data from many material families, ML-based approaches can learn novel descriptors and detect deviations from scaling to predict catalytic activity across compositional spaces where conventional heuristics do not work.

## 3 The machine learning toolkit for materials informatics

Machine learning is a set of disruptive statistical methods in which predictive models use input data to enable computational algorithms to search for non-linear correlations between material properties and catalytic activities. These models enable the potential to attain Density Functional Theory-level accuracy at whole orders of magnitude reduced and cheaper computational cost, thereby establishing ML as a staple in accelerated electrocatalyst discovery (Chen et al., 2025; Wang X. et al., 2025).

### 3.1 The catalyst data ecosystem: sources and curation

Everything a machine learning model can do, in terms of prediction, depends on the training data, its amount, quality, and variety. The electrocatalysis data ecosystem consists of two primary contributions: one computational (mostly density functional theory calculations) and the other experimental metrics such as electrochemical efficiency and material characterisation measurements. These diverse and multi-fold data streams can be synthesized and organized in a manner that helps systematically accustom ML models to quantitative structure–property relations, which are subsequently employed for intelligently steering catalyst design (Jiang et al., 2024).

**FIGURE 4:**
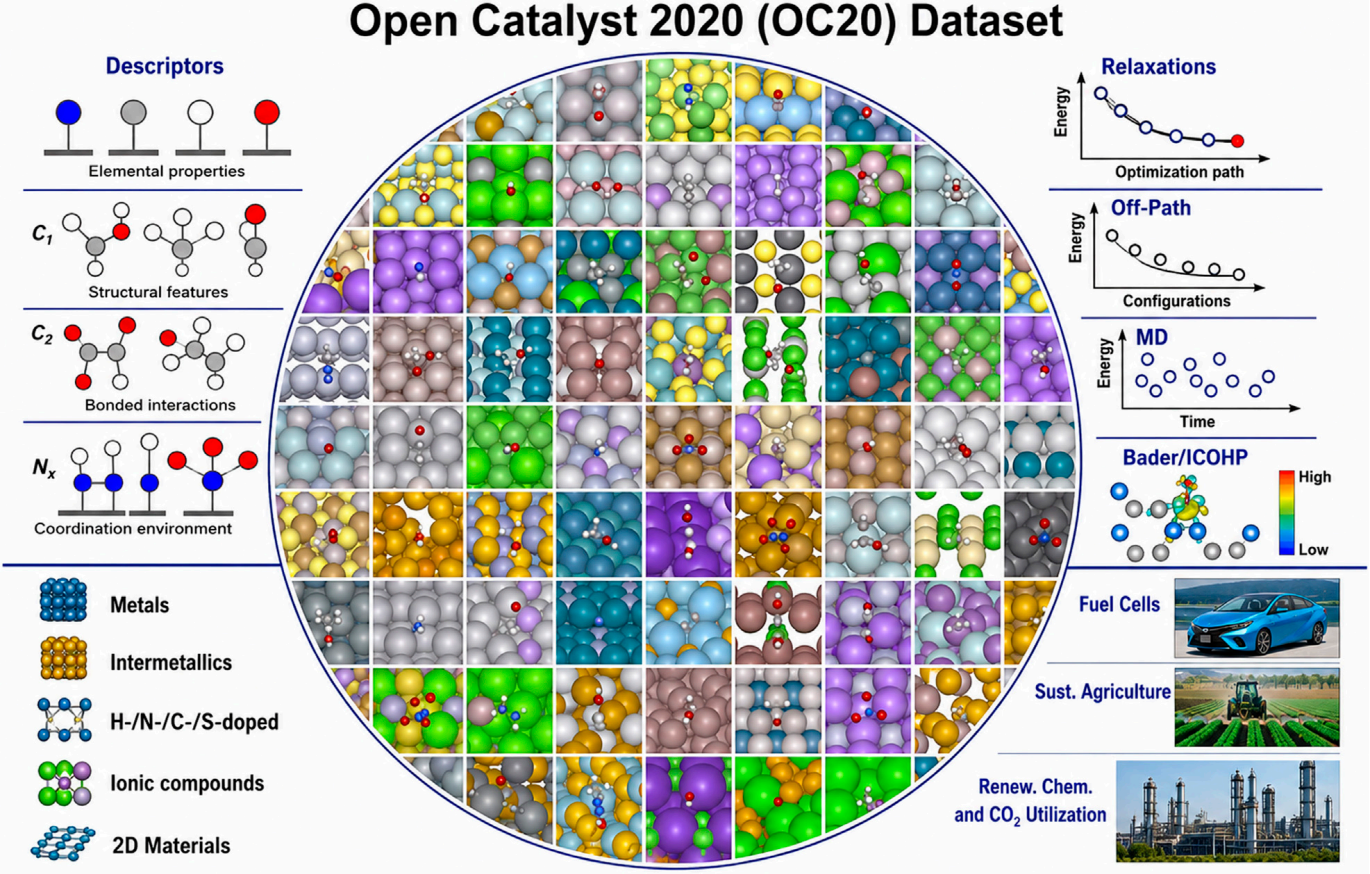
Schematic representation of the Open Catalyst 2020 (OC20) dataset, illustrating its compositional diversity, descriptor space, and atomistic simulation workflows used for training machine learning models in catalysis(Chanussot et al., 2021).

With a focus on high-throughput computational methods, this data landscape has changed rapidly. An example is the Open Catalyst 2020 dataset (Chanussot et al., 2021), which comprises around 1.3 million DFT relaxations on a plethora of materials, surfaces and adsorbates: several orders of magnitude more training examples than was attainable even recently. Its successor, OC22, massively extends this dataset to oxide materials, comprising 62,331 DFT relaxations (nearly ten million single-point calculations), and is designed specifically for the oxygen evolution reaction (OER). This allows atomist machine learning models to be trained with a scale hitherto impossible, sampling applied chemical and compositional diversity spanning elemental combinations of the periodic table. Such large-scale catalyst databases span a wide variety of materials classes, adsorbates and atomistic simulation outputs, as shown in Figure 4.

These datasets underpin much of modern data-centric catalysis and enable machine-learning models to generalize across broad regions of chemical space, making predictions on catalytic activity at scale. However, real experimental data are still hard to feed. Diverse synthesis/characterisation regions and protocols lead to heterogeneous reporting methods, limiting the production of harmonised, machine-readable databases. Examples of such datasets would have many features of (1) the catalyst, including composition, morphology, crystal structure and surface area as well as accurate electrochemical measurements for (2) properties like overpotential, exchange current density, Tafel slopes and stability metrics (Ding R. et al., 2024; Li et al., 2023). Importantly, these datasets need to include both positive and negative outcomes since biases in the published literature towards the positive also lead to systematic bias in models, which impacts their generalizability (Xu S. et al., 2024).

This Table 2 catalogs five large-scale computational databases that underpin modern ML, training: Open Catalyst 2020/2022, Materials Project, NREL, Database, and QMOF., it specifies dataset size, materials covered, target properties (adsorption energies, band gaps, Pourbaix diagrams), and key references. By quantifying the scale (e.g., ∼1.3 million DFT, relaxations for OC20) and diversity (oxides, MOFs, semiconductors), the table illustrates why these resources enable generalizable ML, models. It directly follows the text’s discussion on high-throughput data and serves as a practical reference for readers seeking training data.

**TABLE 2:** Summary of key DFT-based datasets for electrocatalyst ML training (Back et al., 2021; Chanussot et al., 2021; Correa-Baena et al., 2018; Liu et al., 2023).

Dataset name	Size and scope	Materials covered	Target properties	Key reference
Open Catalyst 2020 (OC20)	∼1.3 million DFT relaxations	Metals, alloys, intermetallics, surfaces	Adsorption energies, forces, relaxed structures	Chanussot et al. (2021)
Open Catalyst 2022 (OC22)	62,331 DFT relaxations (∼10M single-point calculations)	Oxides, perovskites, high-entropy oxides	OER intermediate adsorption, oxygen binding	Tran et al. (2023)
Materials Project	>150,000 inorganic crystals	Bulk crystalline materials	Formation energies, band structures, Pourbaix diagrams	Jain et al. (2013)
NREL Materials Database	>1.4 million DFT calculations	Semiconductors, oxides, chalcogenides	Band gaps, formation energies, electronic structure	Stevanović et al. (2012)
QMOF Database	∼20,000 metal-organic frameworks	MOFs with diverse metal nodes	Band gaps, pore volumes, gas adsorption	Rosen et al. (2021)

The solutions to address such challenges are strong data architectures and stringency in data curation standards. With the help of Natural Language Processing (NLP) and Pattern Recognition technique, more recently, published documents have been mined to retrieve the outcomes of synthesis actions, characterization, performance metrics, etc (Suvarna et al., 2023). Standardized internal representations of data (e.g., MDL files, linear notation (SMILES and InChI)) make it readily accessible for algorithmic analysis and reproducibility (Grajciar et al., 2018). These efforts are warranted because, other than an increasingly rich and growing high-quality *in situ* and operando data set that is used to train various AI strategies in heterogeneous catalysis (Bowker et al., 2022; Chen et al., 2023).

#### 3.1.1 Challenges in catalyst data quality, bias and reproducibility

The success of any machine learning model ultimately hinges on the quality, diversity, and reliability of its training data. Although the number of available computational databases of catalysts has increased significantly, there are still problems that limit the generalizability of the models. An important issue is the imbalance of the datasets. Successful catalysts with high catalytic activities have been reported in published literature, but unsuccessful experiments are rarely reported. This publication bias leads to an overestimation of catalyst performance and diminishes the capacity of machine learning models to discover genuinely new materials. Another significant limitation arises from variations in the experimental protocols. Often, electrochemical measurements are performed with varying electrolyte compositions, pH values, temperatures, catalyst loadings, and electrode preparation techniques. Reported overpotentials, Tafel slopes, and stability values may therefore not be directly comparable, leading to systematic uncertainties in machine learning models.

In addition, there are intrinsic uncertainties due to the approximations in the exchange-correlation functionals, the finite size of the supercell, the convergence thresholds, and the assumptions about surface reconstruction made with DFT-generated data sets. While DFT provides physically relevant descriptors, these approximations carry over into machine-learning predictions and must be considered in uncertainty analysis. Recent advances in uncertainty-aware neural networks, Bayesian optimisation, Monte Carlo dropout, Gaussian Process Regression, and ensemble learning provide probabilistic confidence intervals in addition to catalyst predictions. This type of method would allow better screening of catalysts, separating more confident predictions from more uncertain extrapolations.

### 3.2 Featurization: representing catalysts for ML models

A good featurization encodes complex physicochemical properties of the catalysts in a machine-readable format, with implications to both predictive power and interpretability of any models trained on top (Mou L. et al., 2023; Mou T. et al., 2023). It involves selecting (or training) descriptors that capture critical information about structural, electronic, and thermodynamic features, ranging from elemental properties and structural motifs to computed electronic band structures and adsorption energies. The method uses molecular descriptors (indicators of the structure) to describe a catalyst’s properties and relate that experimental behaviour with fundamental structural characteristics (Escayola et al., 2024). These encompass the simplest kind of compositional features, atomic numbers, electronegativities, ionic radii, and more complex features that represent coordination environments, bond distributions and electronic configurations. In periodic systems, the atoms are represented as nodes, and the chemical bonds are represented as edges on a crystal graph, enabling Graph Neural Networks (GNNs) to learn directly from the structure of the crystal graph without hand-crafted features (Motagamwala et al., 2018).

**FIGURE 5:**
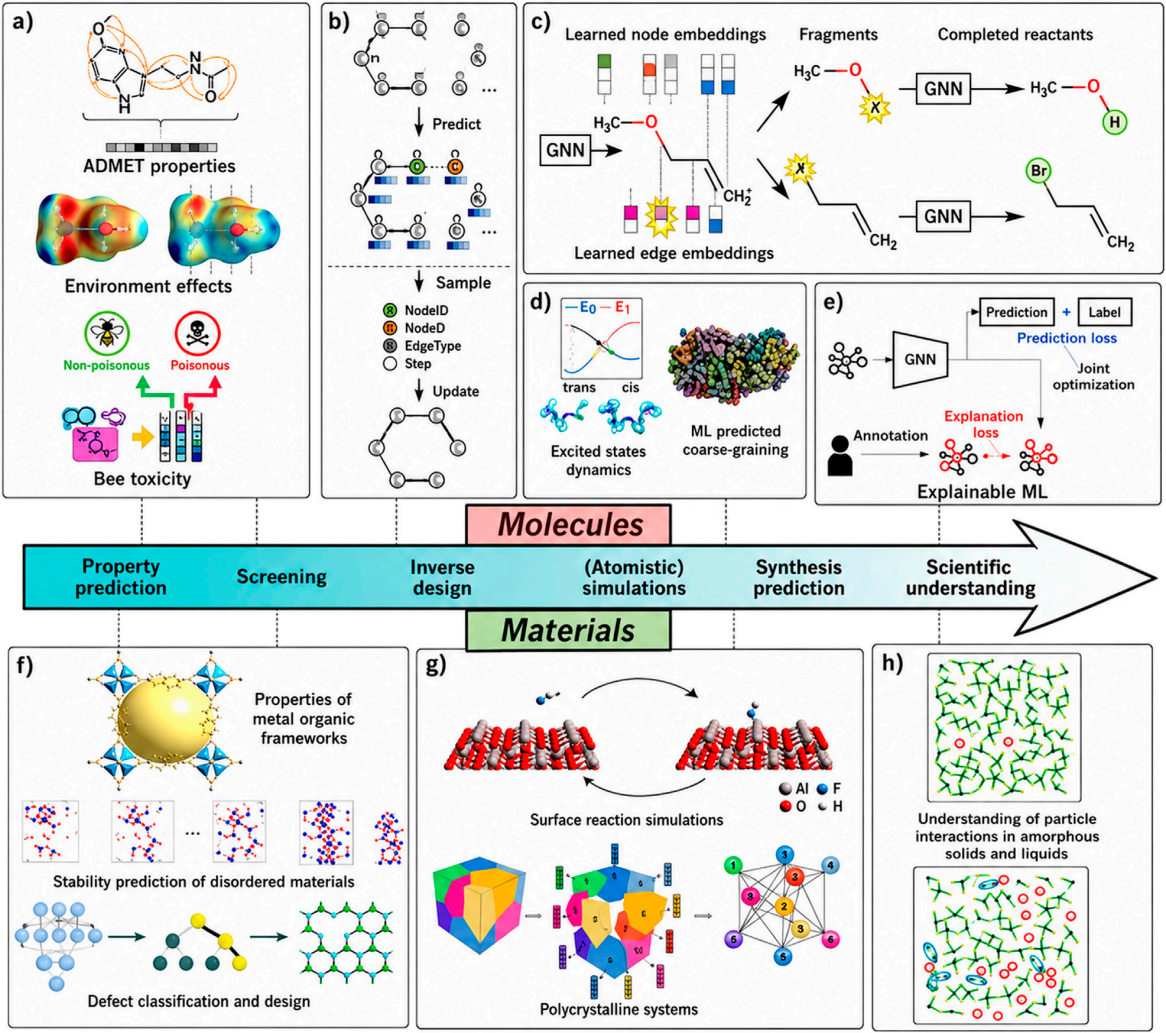
Applications of graph neural networks in molecular and materials informatics for catalyst discovery and property prediction. **(a)** Prediction of molecular properties including ADMET behavior, environmental interactions, and toxicity assessment. **(b)** Iterative graph-based molecular optimization and screening workflow. **(c)** Learning of node and edge embeddings for reaction completion and molecular fragment generation. **(d)** GNN-assisted excited-state dynamics and machine learning-driven coarse-grained simulations. **(e)** Explainable graph neural network framework for interpretable predictions and scientific understanding. **(f)** Stability prediction and defect classification in metal–organic frameworks and disordered materials. **(g)** Surface reaction simulations and prediction of polycrystalline material behavior using graph-based representations. **(h)** Atomic-scale understanding of particle interactions in amorphous solids and liquids through GNN-enabled modeling (Reiser et al., 2022).

A detailed summary of electrocatalyst-discovery applications of graph neural networks (GNNs) is shown in Figure 5. The figure illustrates the GNN architecture’s ability to address several challenges in property prediction, virtual screening, inverse materials design, atomistic simulations, synthesis optimisation, and scientific interpretability. The molecular-level learning tasks of predicting ADMET characteristics, reaction completion, explainable AI, and coarse-grained simulations are emphasized in (a–e), while (f–h) show applications to crystalline, polycrystalline, and amorphous material systems. Overall, this figure highlights the ability of GNNs to efficiently capture complex structure–property relationships directly from atomic environments, making them promising tools for structure-aware machine learning approaches to accelerated catalyst discovery and materials optimisation. A crystal graph representation atom based is shown, with atoms represented as nodes and chemical bonds as edges. This graph structure is then given as an input to graph neural networks (GNNs) to learn atomic environments and predict properties without hand crafted descriptors. The figure pictorially illustrates how the GNNs account for local coordination, bond order and periodicity–which will be further discussed later in the paper in relation with the structure-aware ML for electrocatalysis.

It is worth noting that the optimal featurization strategy depends heavily on the material class and the specific catalytic reaction. In the case of single-atom catalysts, attributes that describe not only the metal centre and its coordination environment but also properties of the support become indispensable (Wang X. et al., 2025). The immense compositional space poses unique challenges for high-entropy alloys, necessitating new methods that can represent local chemical environments and their distributions. Two main strategies have been developed: the “direct” prediction from unrelaxed structures using hand-crafted features or end-to-end graph networks, and the “iterative” prediction through machine learning potentials, which only requires a guide for structural relaxation until energy calculation after adsorption (Wang J. et al., 2025).

GPH convolutional networks have been shown to excel at learning representations directly from atomic structures. These models thus learn to capture a chemical environment responsible for catalytic activity without explicit feature engineering, by passing information through neighbour graphs. Training such models on diverse datasets of materials containing a range of adsorbates and surface terminations is, however, far from trivial in terms of their generalizability (Kolluru et al., 2022). Methods such as Surf Graph circumvent these limitations by employing subgraph representations that describe adsorbate chemical environments, allowing the balance of end-to-end feature learning with chemical intuition in characterizing local interactions essential for catalysis (Ghanekar et al., 2022).

Because learned representations are “black box,” interpretable featurization strategies that sacrifice some physical intuitions are often used. Descriptor-based methods, e.g., using physically meaningful features (e.g., d-band centre, coordination numbers, or adsorption energies), are interpretable but tend to have lower predictive power than learned representations. Physics-informed machine learning merges the (while also without loss of generality non-linear) ability of machine learning to learn solid structural relationships from data with our physically-domain knowledge of the governing processes through the imposition of our domain knowledge in physical processes at either model architectures or loss functions so that we can guide our learner toward physically meaningful solutions while offering it competent feature descriptors (Omranpour et al., 2025).

Table 3 shows an overview of four representative featurization strategies adopted in electrocatalysis ML: 1) the adsorption energies of the molecules in the dataset (OC20/OC22), 2) featurization of the crystal graph of the molecules (GNN inputs), 3) hand-crafted features such as d band centre (descriptors-based featurization). For every, the table provides key features captured, material classes covered and primary applications (HER/OER screening, structure–property prediction). It provides insights into the trade-off between end-to-end graph learning and interpretable descriptor approaches, and continues to drive home the point that the choice of featurization will affect metrics such as predictive performance and model transparency.

**TABLE 3:** Overview of key machine learning datasets and featurization strategies.

Dataset/Featurization	Description	Key features	Material classes covered	Primary application	Ref.
Open Catalyst 2020 (OC20)	∼1.3 million DFT relaxations of materials, surfaces, and adsorbates	Adsorption energies, forces, bulk and surface structures	Metals, alloys, intermetallics	HER, CO_2_ reduction, general catalysis	Chanussot et al. (2021)
Open Catalyst 2022 (OC22)	62,331 DFT relaxations (∼10M single-point calculations)	Oxide surfaces, OER intermediates, oxygen adsorption	Oxides, perovskites, high-entropy oxides	OER, oxygen electrocatalysis	Tran et al. (2023)
Crystal Graph Representations	Atoms as nodes, bonds as edges in graph neural networks	Coordination environment, local geometry, bond order	Crystalline materials, alloys, oxides	Structure–property prediction, GNN-based screening	Peng et al. (2022)
Descriptor-Based Featurization	Hand-crafted features (e.g., d-band centre, electronegativity, ionic radius)	Elemental properties, electronic structure, thermodynamic stability	Alloys, high-entropy alloys, SACs	Interpretable models, descriptor discovery	Mou L. et al. (2023)

### 3.3 Algorithmic landscape: from simple models to deep networks

Building a machine learning architecture that is both accurate and easy to understand requires a trade-off among accuracy, interpretability, computational resource costs, and data requirements. The scope of this algorithm ranges from linear models to sophisticated deep learning architectures, each with its own merits for specific tasks and materials systems. Some of the initial methods to predict catalyst properties employed linear regression, support vector machines, and decision trees. These models are not too complicated, not too complex, are not too costly, and are attractive in terms of downstream speed and understandability (Li et al., 2023). This suite of features provides accurate classification of solid-solution phases of HEAs, with varying lengths of descriptor ensembles, and support vector machines can work with highly complex decision boundaries in the higher-dimensional space of the descriptors. The kernel ridge regression is a generalised linear technique that transforms the vector of inputs into a higher dimensional feature space, to represent nonlinearities in convex optimisations that define locality of extrema and minimise risk. But as the datasets became larger and more complex, so did the techniques. Random forests leverage predictions from hundreds of decision trees, which prevents overfitting when ranking feature importance (i.e., dictating which descriptors have the most influence on catalytic performance). Gradient boosting machines such as XGBoost, LightGBM, and CatBoost improve iteratively upon their predictions by focusing on samples that were previously mis predicted and have achieved state-of-the-art performance for moderate-sized training sets (Chen et al., 2025) over tabular data.

Table 4 a technical comparison of five state-of-the-art ML interatomic potentials: SchNet, DimeNet++, GemNet, MACE, and Allegro. For each, it specifies architecture type, key features (e.g., rotational invariance, many-body expansion), typical accuracy (MAE relative to DFT), computational cost, and best-suited applications (e.g., high-entropy alloys, oxide surfaces, large-scale interfaces). This table complements the algorithmic discussion by focusing specifically on potentials used for atomistic simulations, helping researchers select the right tool for tasks like adsorption energy prediction or molecular dynamics.

**TABLE 4:** Comparison of common ML interatomic potentials for electrocatalysis simulations. Source.

Potential name	Architecture type	Key features	Typical accuracy (MAE)	Computational cost	Best suited for	Key reference
SchNet	Continuous-filter CNN	Rotationally invariant, end-to-end learning	∼0.05 eV (energies), ∼0.05 eV/Å (forces)	Medium	Molecular dynamics, adsorption energies	Schütt et al. (2018)
DimeNet++	Directional message passing	Includes angle information, 3-body interactions	∼0.03 eV (energies)	High	Oxide surfaces, complex coordination environments	Gasteiger et al. (2022)
GemNet	Geometric message passing	4-body interactions (dihedral angles)	∼0.02 eV (energies)	Very high	Large-scale interface simulations	Gasteiger et al. (2024)
MACE	Atomic cluster expansion	Many-body expansion, linear scaling	∼0.01 eV (energies)	Medium	High-entropy alloys, disordered systems	Batatia et al. (2023)
Allegro	Local equivariant transformer	Strictly local, E(3) equivariant	∼0.02 eV (energies)	Medium-High	Large systems (>10,000 atoms)	Musaelian et al. (2022)

**FIGURE 6:**
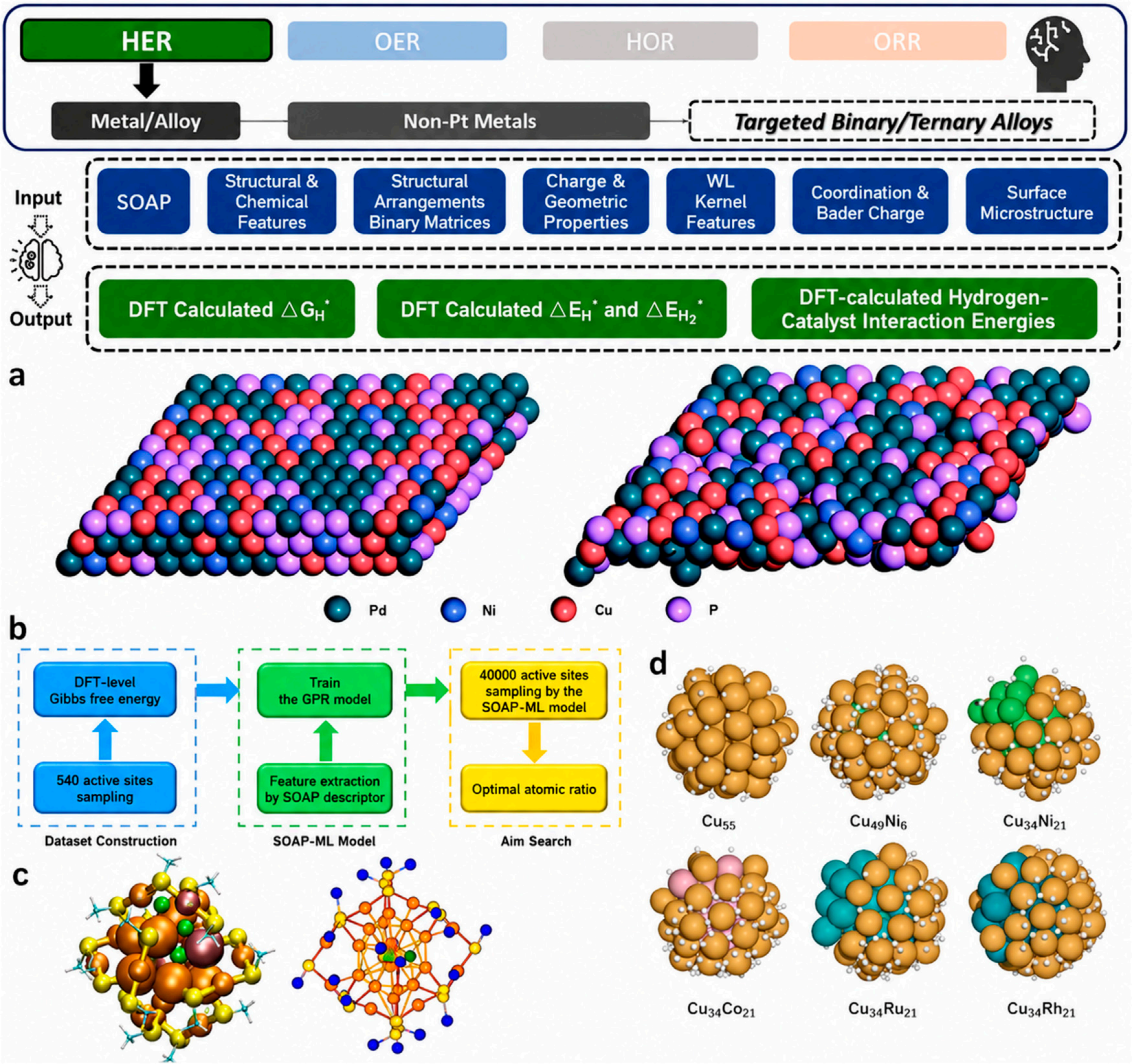
Alloy optimization and screening for hydrogen evolution reaction (HER). **(a)** Process of descriptor generation and machine learning-based prediction of the energetics of hydrogen adsorption in binary and ternary alloy systems, including descriptors related to structure, electronic structure, and coordination. **(b)** Development of a machine-learning-based model to find optimal adsorption sites and atomic structures of Pd-Ni-Cu alloy catalysts. **(c)** A representation of doped catalyst structures based on graphs that can be used to learn local atomic environments and their adsorption behaviour. **(d)** For multi-metallic Cu-based alloy nanoclusters, with near-thermoneutral hydrogen binding energies, predict optimal hydrogen adsorption sites (Ding R. et al., 2024).

Figure 6 shows representative machine learning workflows used for the rational design and optimization of alloy electrocatalysts for the hydrogeneration reaction. They show how the combination of descriptor engineering, graph-type structural representations, and machine learning models with the help of soap can facilitate the identification of catalytically active alloy compositions and adsorption sites at a faster pace. Subfigure (a) illustrates the linkages among structural description, geometric structure, and electronic description and the combination of these with density functional theory-based adsorption energetics. Subfigure (b) illustrates an iterative active-site optimisation process for identifying active sites in Pd-based alloys. The remarkable ability of graph representations and machine learning-assisted screening to encode the local coordination environment and to identify favourable adsorption configurations in complex multi-metallic catalyst systems is further highlighted in subfigures (c) and (d). In general, the figure illustrates the growing importance of data-driven approaches to more rapidly discover alloys for electrocatalysis and to minimise the time spent on full trial-and-error experiments.

A comparative chart positioning common ML algorithms (linear models, random forests, GNNs, PINNs, generative models) along three axes: interpretability, computational complexity, and predictive capability. It helps readers decide which algorithm class suits a given task–fast, interpretable models for descriptor mining versus high-capacity deep networks for adsorption energy prediction–while acknowledging the inherent trade-offs. Now, deep learning architectures have taken over for advanced material representations. Convolutional neural networks, which emerged as a breakthrough for image recognition tasks, lend themselves to periodic systems through the adaptation of electron density maps or charge densities generated from DFT calculations. Graph neural networks (GNNs) such as message-passing neural networks, graph convolutional networks, and graph attention networks operate directly on atomic structures and learn to propagate information through neighbour graphs in order to predict properties at a global, atomic, or bond level (Resasco et al., 2022). During this period, ML has emerged as a powerful tool to model heterogeneous catalysis, particularly in the form of so-called neural network potential energy surfaces, which capture the local chemical surroundings that determine catalytic activity and yield homogenous DFT-like accuracy for adsorbate energies over replicates of slightly different surface structures. In this domain, the Open Catalyst project has accelerated progress significantly by providing standardized benchmarks and large-scale datasets. Graph neural network architectures—CGCNN, SchNet, DimeNet++, and later variants—systematically enhanced energy and force predictions, where larger models outperformed smaller ones (Chanussot et al., 2021). However, this brute-force data-scaling approach may still require further advances in architecture to accomplish chemical accuracy (≈0.05 eV) of adsorption energies across disparate materials.

**FIGURE 7:**
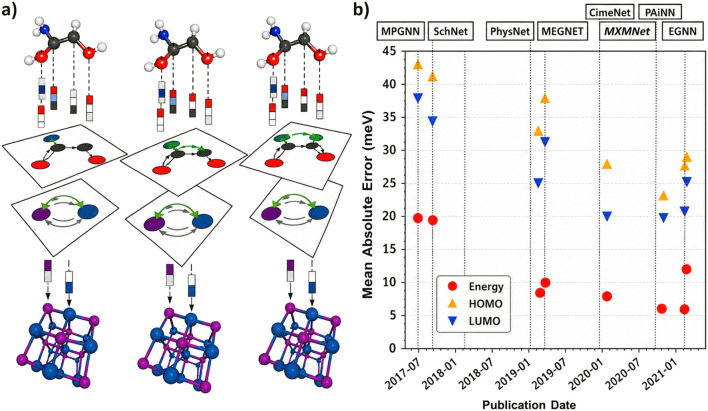
Use of message passing and prediction accuracy for molecular and crystalline materials modelling using graph neural networks. **(a)** Schematic picture of the message-passing operation in graph neural networks, showing the iterative exchange of information between neighbouring atomic environments in molecular and crystalline systems. **(b)** Comparative evaluation of the performances of state-of-the-art graph neural network architectures on predicting molecular electronic properties like energy, HOMO and LUMO energies (Reiser et al., 2022).

Graph neural networks (GNNs) have a fundamental operating principle and predictive ability in materials informatics and electrocatalysis applications, as shown in Figure 7. Subfigure (a) illustrates the message-passing model employed, in which the local atomic environment is represented as an atomic feature and propagated from neighbouring nodes and edges to model a chemically meaningful representation of the local environment. This allows GNNs to learn structural, geometric, and electronic interactions directly from atomic configurations without requiring manually designed descriptors. To demonstrate this evolution, Figure (b) shows the predictive accuracy of several advanced GNN models on several molecular properties, where the accuracy of graph-based deep learning models keeps improving as they become more complex. The structures highlight the increasing relevance of message-passing neural networks as an effective method for catalyst property prediction and materials discovery, given their ability to predict catalyst properties from the catalyst’s structure and composition.

Example of diagram of a GNN message passing framework. Represents how neighboring atoms (such as types of elements, bond distances) will be recursively added and modified to generate predictions of properties at the atomic level or for the entire molecule. The figure uncovers the “black box” aspect of GNN by clearly showing the information flow, and it is directly connected with the explanation provided in the text regarding how GNNs learn chemical environments. The model-driven design for catalysis is one of the leading new applications of ML in the field of catalyst design. Learning the underlying distribution of crystal structures from training on such stable structures allows generative models, such as variational autoencoders, GANs, and diffusion models, to synthesize materials with desired properties without the need to forward screen (Zhou et al., 2026). These models, when paired with property predictors and optimisation algorithms, can suggest new compositions and structures that meet multiple design targets (activity, stability, cost, and synthesizability).

In Table 5, we summarize the key families of ML methods that can be applied to electrocatalyst design: interpretable models, such as linear regression and decision trees; ensemble models, such as the random forest and the XGBoost; graph neural networks, such as CGCNN and SchNet; generative models, such as the variational autoencoder (VAE), the generative adversarial network (GAN), and diffusion models; and physics-informed neural networks (PINNs). The table shows the strengths and limitations of each class: interpretability, accuracy, inverse design and data demand, opacity and complexity, respectively. For readers, it is a convenient tool to follow the path of the choices that are given in the body of the text starting from quick (fast) descriptor mining and reaching the black box deep learning.

**TABLE 5:** Machine learning algorithms for electrocatalyst design source (Chen et al., 2025; Li et al., 2023; Wang S.-H. et al., 2021; Zhou et al., 2026).

Algorithm class	Representative models	Key strengths	Limitations	Typical applications
Interpretable Models	Linear regression, decision trees, symbolic regression	High interpretability, low data requirements, fast training	Limited capacity for complex, non-linear relationships	Descriptor discovery, design rule extraction
Ensemble Methods	Random forest, XGBoost, LightGBM, CatBoost	Good balance of accuracy and interpretability, handles tabular data well	Less effective for complex structural data	Property prediction, feature importance ranking
Graph Neural Networks (GNNs)	CGCNN, SchNet, DimeNet++, GemNet, MEGNet	Learns directly from atomic structure, high accuracy	Black-box, high data demand, computationally intensive	Adsorption energy prediction, virtual screening
Generative Models	VAE, GAN, diffusion models, LLMs	Inverse design, *de novo* structure generation, novelty exploration	Requires careful conditioning, synthesizability gap	New alloy/perovskite generation, multi-objective design
Physics-Informed Neural Networks (PINNs)	PINN variants, concept bottleneck models	Embeds domain knowledge, physically consistent predictions	Complex architecture design, training challenges	Stability forecasting, Pourbaix diagram prediction

When employing one algorithm in predictive scenarios over another, in addition to predictive performance, one should consider the need for interpretability, computational costs, and uncertainty quantification. On one extreme there are simple models or decision trees that are easy to follow and can only capture limited concepts, and on the other extreme there are high-capacity deep networks that attain to higher accuracy, but a corresponding loss of transparency. PINNs directly embed the physics knowledge into the architecture or loss function of the network, which is how they can be made interpretable without compromising deep learning capabilities (Wang S. et al., 2021). Similarly, Bayesian methods and ensemble approaches yield natural uncertainty measures which can help guide experimental testing to promising candidates, provide a way for the researcher to gain confidence in the model at any time, and help inform regions of chemical space that may lead to new opportunities. Note that there is now a rise in many architectures, and therefore, all other parts of the training process. The most effective methods combine several of the above, such as using graph neural networks to encode the structure, transferring knowledge from related data sets, using active learning to gradually select most informative examples as training data, and employing multi-objective optimisation to address conflicting design objectives. The immense toolbox can be used to search and optimise electrocatalysts for sustainable energy applications considerably faster when used judiciously.

### 3.4 Critical comparison of machine learning approaches for electrocatalyst discovery

In addition to traditional reviews that primarily provide an overview of algorithmic advances, assessing the practical viability of various machine learning methods for catalyst discovery is crucial. Classical machine learning algorithms like Random Forest (RF), Support Vector Machines (SVM) and Gradient Boosting Machines (GBM) typically work well with relatively small data sets, because they have a good tolerance of overfitting and can identify important descriptors that govern catalytic activity. All those techniques, however, rely heavily on artificially designed descriptors and do not capture complex atomic interactions in heterogeneous catalyst systems. To address many of these challenges, deep learning models, notably Graph Neural Networks (GNNs), can be trained directly from crystal structure, thereby avoiding the need for handcrafted descriptors to learn atomic environments. They are good at predicting adsorption energies and catalytic activities across large chemical spaces due to their ability to model local coordination, electronic interactions, and surface geometries. However, GNNs place significant demands on data size, training time, and computational resources. Moreover, they are black-box models: although they perform well at prediction, there is not necessarily a scientific understanding of this type of model. Combining electrochemical constraints into model training by physics-informed neural networks or hybrid ML-DFT frameworks can offer an attractive compromise. These methods enhance extrapolation beyond the training sets, reduce data requirements, and increase physical consistency. However, their implementation remains computationally challenging and must be carefully formulated with physical constraints governing it.

### 3.5 Explainable artificial intelligence for catalyst design

With the growing complexity of machine learning models, the importance of model interpretability is increasing as well, alongside prediction accuracy. Explainable Artificial Intelligence (XAI) offers tools to identify the physicochemical factors underlying model predictions and allows one to gain scientific insight beyond the mere predictions themselves. Methods that assign importance to each feature, such as SHAP (SHapley Additive exPlanations) and Local Interpretable Model-agnostic Explanations (LIME), quantify the relative importance of features such as d-band centre, electronegativity, coordination number, adsorption energy, and crystal symmetry in determining the predicted catalytic performance. These approaches allow researchers to determine the main descriptors of the catalyst and to check the machine-learning predictions against known electrochemical principles. GNNExplainer and attention visualisation are methods used to identify catalytically active atomic environments responsible for predicted adsorption energies in GNNs. These methods can greatly enhance the reliability of deep learning models and help to design catalysts more rationally by uncovering new structure–property relationships. The emerging symbolic regression methods also enhance interpretability by developing explicit mathematical models that relate catalyst descriptors to electrocatalytic activity. These interpretable models could someday connect the dots between the traditional descriptor-based approach to catalyst design, and modern deep learning.

## 4 Strategic applications of ML across the catalyst lifecycle

In the case of electrocatalyst R&D, the machine learning techniques are especially appropriate as they can be used to guide the process of discovery, mechanistic understanding, synthetic realisation, and performance benchmarking at all the stages of the overall process. This integration is aimed at promoting rational design and utilization of highly efficient, durable catalysts for important electrocatalytic processes (Guo et al., 2024; Salami et al., 2025).

A representative list of six electrocatalyst discoveries using ML is provided in Table 6, and these discoveries have been successfully validated in the lab. The entries include a description of the catalyst system, target reaction (HER/OER), ML approach (random forest + Bayesian optimisation, GNN + high-throughput screening, etc.), key finding, measured performance (overpotential, stability, mass activity), and citation. This table, which appears just before Section 4.1, shows the practical impact of ML in real applications, from high-entropy RuO_2_ (1500 h stability) to ternary Pt alloys (8× higher activity than Pt/C), thereby rooting the ensuing strategic discussions in tangible applications.

**TABLE 6:** Representative ML-driven electrocatalyst discoveries with experimental validation.

Catalyst system	Target reaction	ML approach	Key finding	Experimentally validated performance	References
NiFe-LDH with dopants	OER (alkaline)	Random forest + Bayesian optimization	Identified Cr and Mo as optimal co-dopants	Overpotential ∼220 mV @ 10 mA cm^−2^ , stability >500 h	Xu S. et al. (2024)
High-entropy RuO_2_	OER (acidic)	Graph neural network + high-throughput screening	Multi-cation synergy prevents Ru dissolution	Overpotential ∼180 mV @ 10 mA cm^−2^ , 1,500 h stability	Qian et al. (2023)
Ternary Pt-alloys (Pt-Pd-Au)	HER (acidic)	Symbolic regression + volcano mapping	Optimal composition: Pt_45_Pd_35_Au_20_	Mass activity 8× higher than Pt/C	Cao et al. (2025)
Perovskite oxides (Ba_x_Sr_1-x_Co_yFe_1-γ_O_3_)	OER (alkaline)	Transfer learning + GNN	Ba_0_._5_Sr_0_._5_Co_0_._8_Fe_0_._2_O_3_ identified as optimal	Overpotential ∼320 mV @ 10 mA cm^−2^	Jiang et al. (2021)
Single-atom catalysts (M-N-C, M = Fe, Co, Ni)	HER (all pH)	Descriptor-based ML + DFT screening	Fe-N_4_-C with axial OH ligand predicted	ΔG_H ≈ −0.05 eV, near-Pt activity	Li (2022)
High-entropy alloys (Cr-Mn-Fe-Co-Ni)	OER (alkaline)	Multi-objective Bayesian optimization	Equimolar composition with 5% Mo addition	Overpotential ∼260 mV @ 10 mA cm^−2^	Xu S. et al. (2024)

### 4.1 Activity prediction and virtual screening

The most developed use of these machine learning techniques is the prediction of catalytic activity from materials descriptors; normally, a compositional database contains millions of potential materials, and high-throughput screening of these is only possible if they can be predicted as catalysts. Instead of having to analyze and characterize several thousand materials experimentally, the ML models allow virtual screening that is, consider millions of candidates *in silico*, but focus experiments on the most promising ones (Schrier et al., 2023).

**FIGURE 8:**
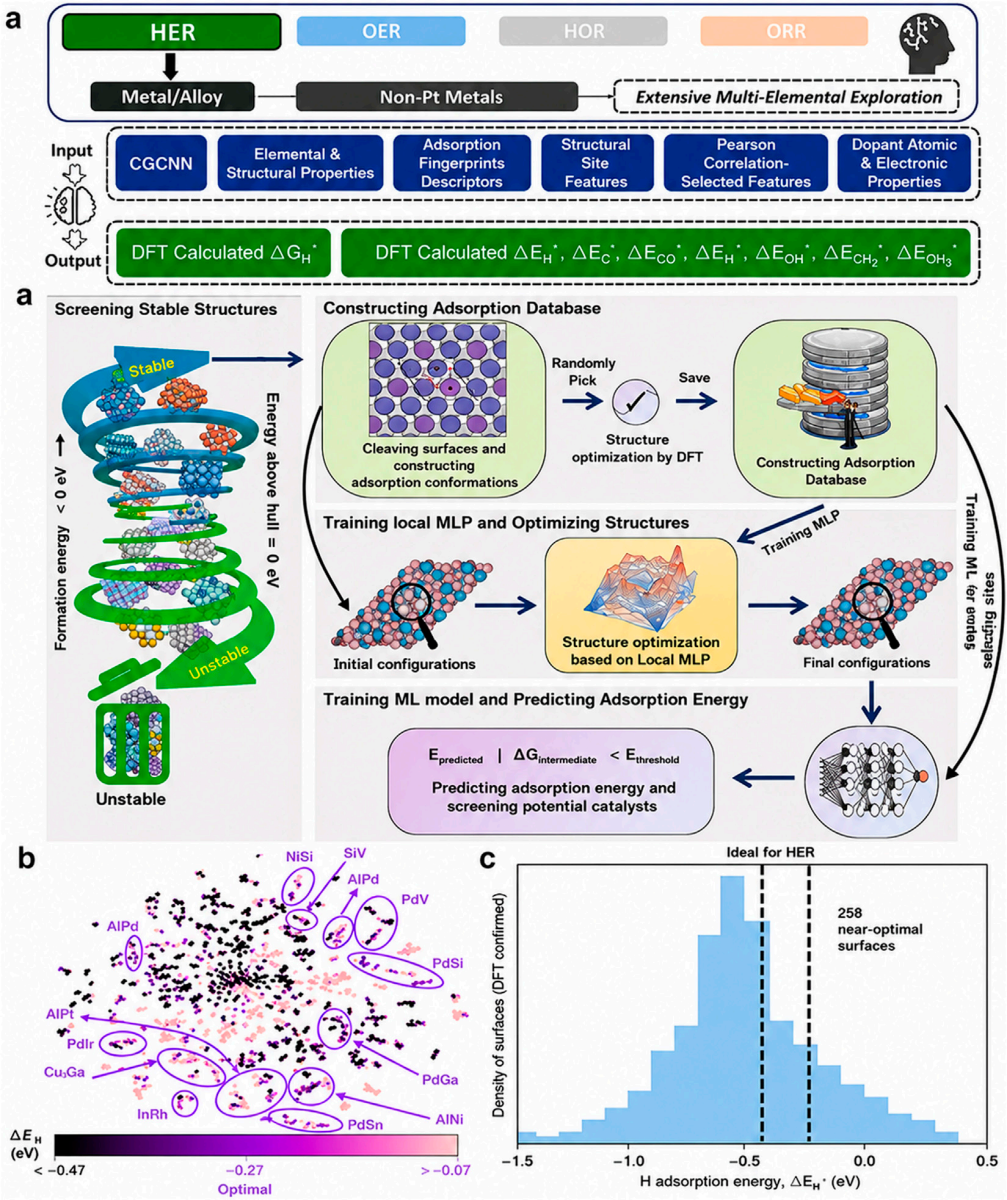
**(a)** Machine learning workflow for multi-element electrocatalyst screening via descriptor engineering, gNN models and density functional theory (DFT) derived adsorption energetics. **(b)** Stable structure selection, adsorption site generation, training adsorption energy machine learning potentials and prediction steps of building a high-throughput adsorption database, and an iterative optimisation pipeline. **(c)** Distributed stochastic neighbours embedding (t-SNE) visualisation of the adsorption-energy landscapes for each alloy surface analysed to identify candidate regions for structures with hypothetical optimal hydrogen adsorption energy. **(d)** Statistical distribution of hydrogen adsorption energies (ΔE_h_) of screened surfaces with identification of near-thermoneutral HER-active catalyst candidates (Ding R. et al., 2024).

As shown in Figure 8, a comprehensive framework for high-throughput screening and optimisation of Hydrogen Evolution Reaction (HER) electrocatalysts using a machine learning approach is presented. A comprehensive framework for high-throughput screening and optimization of Hydrogen Evolution Reaction (HER) electrocatalysts by a machine learning approach is demonstrated in Figure 8. Subfigure (a): Combination of graph neural network architectures, structural descriptors and the adsorption energetics from a density functional theory calculation for quick prediction of catalytic activity for complex multi-element alloy systems. (b) Shows the automated workflow to build adsorption databases and train machine learning potentials to speed up structural optimization and energy calculations of adsorption. The adsorption-energy landscape visualization in (c) shows how dimensionality-reduction techniques can be used to identify clusters of promising catalyst candidates with near-optimal hydrogen adsorption behavior, and (d) statistically maps the distribution of adsorption energies to pick out the thermoneutral HER-active surfaces. Together, the figure showcases a dramatic decrease in computational requirements and thus the speed of exploring the vast compositional space of catalysts by machine learning, allowing the discovery of efficient electrocatalysts.

#### 4.1.1 High-throughput discovery of novel alloys, perovskites, and SACs

The engine behind modern materials discovery is high-throughput computational screening combined with quantum-mechanical approaches. Machine learning (ML) models trained on DFT calculations can predict important properties such as adsorption energies, formation energies, band gaps over compositional spaces so large that full *ab initio* exploration is still a daunting task, intractable by brute-force (Oh et al., 2024). This capability is transformative for high-entropy alloys: ML models traverse the near-infinite combinatorial space of multi-metallic compositions, uncovering candidates that defy conventional scaling relations and display unprecedented activity (Xu W. et al., 2024). Incorporation of computationally verified structural models into active learning workflows reduces the need to rely on purely *ab initio* methods by orders of magnitude (Lan et al., 2023), while predictions are iteratively improved.

ML models predict band edge positions and optical absorption spectra for perovskite oxides to direct the discovery of photocatalysts for solar-driven water splitting (Oh et al., 2024). First-principles calculations revealed that perovskite materials can possess a favourable band gap and align right between the conduction band of water oxidation and the valence band of water reduction for overall water splitting. Likewise, for single-atom catalysts, ML models traverse the expansive design space of metal centres, coordination environments, and supports to deliver hydrogen adsorption free energies at near-Density Functional Theory (DFT) accuracy and identify systems with comparable or superior performance to platinum (Li et al., 2023; Wang C. et al., 2025).

**FIGURE 9:**
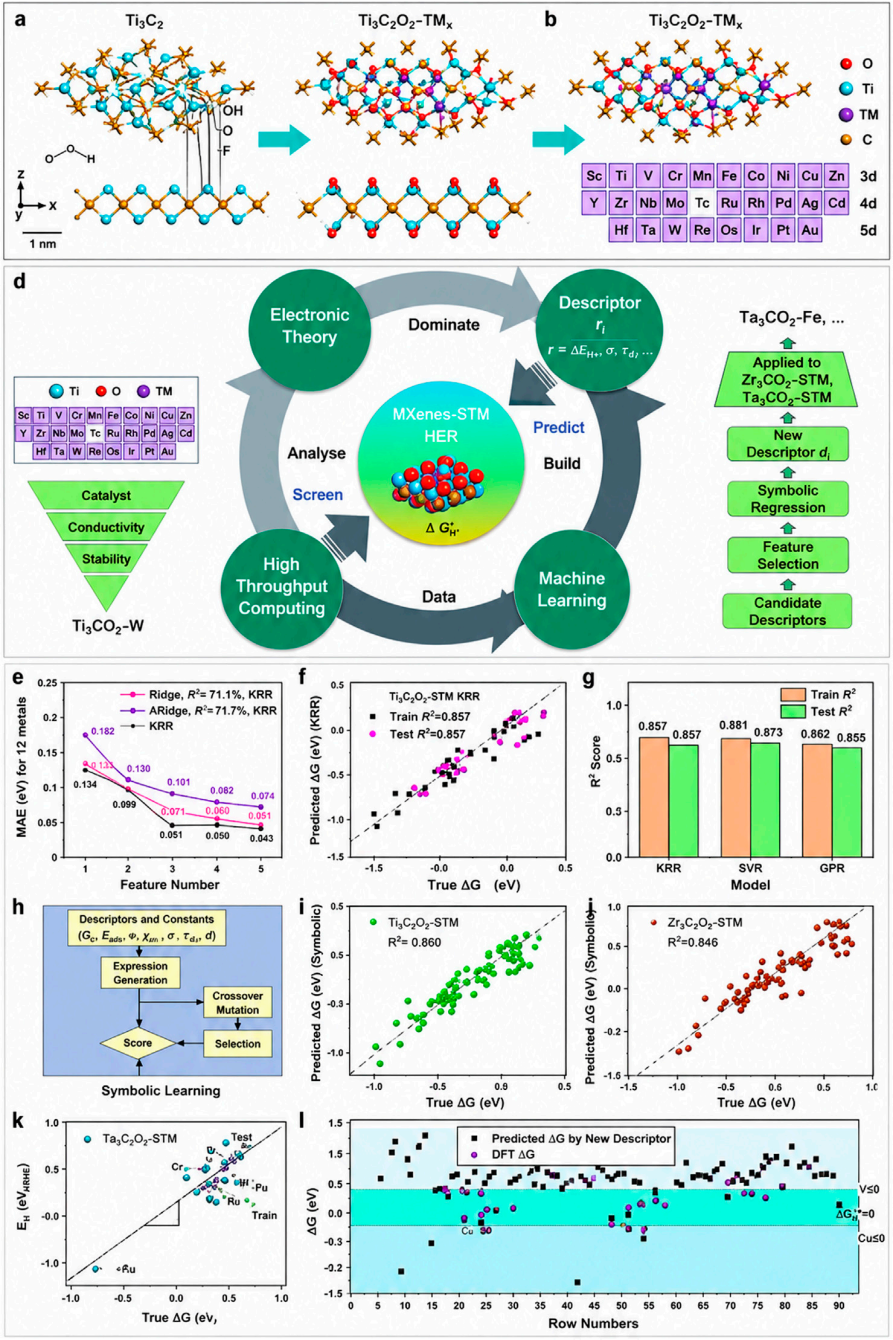
Machine learning-assisted descriptor engineering and screening of MXene-based HER electrocatalysts. **(a–c)** Atomic structures of pristine and transition-metal-modified MXene systems illustrating adsorption-site configurations and compositional engineering strategies. **(d)** Integrated high-throughput computational and machine learning framework for descriptor discovery, catalyst screening, and hydrogen adsorption prediction in MXene electrocatalysts. **(e–g)** Performance evaluation of multiple regression models for predicting hydrogen adsorption free energy (ΔG_h_), including feature selection, correlation analysis, and model validation metrics. **(h)** Symbolic learning workflow for identifying interpretable catalyst descriptors from electronic and structural parameters. **(i–l)** Validation and screening of newly designed MXene catalysts using machine learning-predicted adsorption energetics and descriptor-guided optimization (Li et al., 2023).

The full machine learning approach to the idealized engineering of descriptors and fast discovery of MXene derived electrocatalysts for the hydrogen evolution reaction is illustrated in Figure 9. The pristine and transition-metal-modified MXene systems are engineered to optimize the hydrogen adsorption properties by structural tuning, as shown in subfigures (a–c). The integrated workflow concept is expanded in subsection (d) to include high-throughput computation, electronic structure theory and machine learning to generate descriptors and to screen catalysts. Subfigures (e–g) test the predictive power of several regression models for hydrogen adsorption free energies as a function of the structural descriptors; in subfigure (h), we focus on the use of symbolic regression for deducing interpretable physicochemical descriptors that govern the catalytic activity. Lastly, subfigures (i–l) demonstrate the reliability of the newly derived descriptors in predicting high-performing MXene catalysts with near-optimal hydrogen-adsorption energetics. The figure shows the overall picture of how descriptor-driven machine learning frameworks could increase the speed of rational catalyst design without compromising on the mechanistic interpretability. An ML-assisted screening pipeline is enabled for hydrogen-evolution catalysts. It begins with the engineering of descriptors (d-band centre, electronegativity, etc.), which then go through a workflow that includes model training, prediction of ΔG_H or overpotential, and experimental validation. The figure provides a practical HER example that connects general screening considerations to a concrete case, illustrating the efficient screening of low-dimensional catalysts (e.g., SACs, 2D materials).

#### 4.1.2 Predicting multi-functional activity for overall water splitting

ML can also predict not only the catalytic performance of a single reaction but also materials that are optimal for both HER and OER, meeting the requirements for effective overall water splitting. This necessitates models that maximize multiple catalytic descriptors at once through the identification of bifunctional materials with the ability to operate robustly across different electrochemical conditions encountered at the cathode and anode (Li et al., 2023).

Multi-objective optimization frameworks incorporate independent predictors of HER and OER activity, balancing the trade-off between these two half-reactions to identify compositions that can lead to optimal performance. Such approaches have led to the identification of bifunctionally active transition metal phosphides and chalcogenides, with activities approaching precious metal benchmarks (Ding R. et al., 2024). For example, graph neural networks trained on datasets containing both half-reactions learn joint representations that reflect the underlying electronic descriptors controlling both HER and OER, thereby indirectly enabling prediction of overall water-splitting performance from composition alone.

### 4.2 Stability and durability forecasting

Activity predictions alone are insufficient for practical catalyst development; only long-term stability under realistic conditions can validate a catalyst’s commercial prospects. Recent solutions to this challenge are based on machine learning models that predict degradation mechanisms and durability throughout the catalyst lifecycle.

**FIGURE 10:**
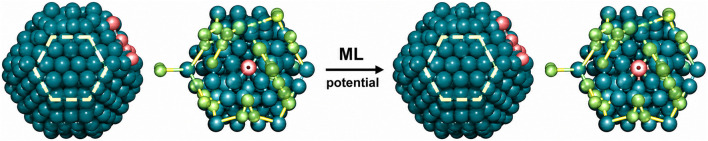
Machine learning-driven stability prediction and compositional optimization of high-entropy alloy electrocatalysts (Ding R. et al., 2024).

Figure 10 illustrates the application of machine learning methodologies for predicting stability, compositional evolution, and electrocatalytic performance in high-entropy alloy (HEA) systems. Subfigure (a) demonstrates the use of machine learning-assisted Monte Carlo simulations to optimize nanoparticle atomic arrangements and identify energetically favorable configurations. Subfigure (b) presents a stability-analysis workflow in which machine learning-derived interatomic potentials are employed to model surface atom dissolution and structural evolution under catalytic operating conditions. Subfigure (c) compares machine learning-predicted onset potentials with experimentally measured values across a ternary Pt–Rh–Sn compositional space, highlighting the predictive capability of data-driven models for screening stable and catalytically active alloy compositions. Collectively, the figure demonstrates how machine learning frameworks can accelerate stability forecasting and compositional optimization in complex multi-element electrocatalyst systems while significantly reducing computational and experimental costs. A schematic of an ML-based framework that predicts degradation pathways–dissolution, surface reconstruction, phase transformation–under operando conditions. It highlights how graph neural networks can learn correlations between crystallography, composition, and dissolution potential, enabling long-term stability forecasts without exhaustive lifetime testing. This figure directly addresses the gap between activity and practical durability.

#### 4.2.1 Modelling dissolution, phase transformation, and surface reconstruction

Harsh electrochemical environments, such as those found in water electrolysis (e.g., under highly acidic or alkaline electrolytes, oxidizing potentials, and elevated temperatures), activate several degradation pathways. As a result, catalyst dissolution leaches active metals into the electrolyte, gradually deactivating the electrode. Active surface structures are phase-transformed to inactive forms. Surface reconstruction enables atomic-level rearrangement, thereby modulating active-site populations and binding energetics. Models using operando characterization data for machine learning can also predict susceptibility to these degradation mechanisms (Zhang H. et al., 2024). Graph neural networks learn the crystallography of a metal oxygen bond and its composition to find associations with dissolution potential. This enables identification of vulnerable sites that are likely to cleave at anodic conditions. In the case of high-entropy alloys, ML models predict how surface composition determines resistance to leaching and passivation (Wang C. et al., 2025). Using direct state inputs, time-series models trained on extensive electrolysis data predict performance trajectories, enabling the identification of failing catalysts long before expensive long-term testing. Most stable catalysts now exhibit extraordinary stabilities, e.g., high-entropy RuO_2_- based systems have managed continuous operation of over 1,500 h at 100 mA cm^−2^ without significant degradation, directly tackling concerns regarding OER stability (Qian et al., 2025) broadly shared by many conventional OER electrocatalysts. Machine-learning (ML) analysis reveals that cooperative interactions among multiple cations stabilise active Ru sites, preventing the over-oxidation and dissolution pathways that rapidly deactivate pure RuO_2_.

#### 4.2.2 Limitations of static machine learning models for long-term stability prediction

While GNNs have been trained on static crystal structures with great success to predict adsorption energies and catalytic activity, long-term catalyst durability is much more difficult. Electrochemical degradation is a dynamic physicochemical phenomenon, which encompasses surface reconstruction, lattice distortion, dissolution of cations, migration of defects, catalyst-support interactions, electrolyte effects, and morphological evolution and requires long operation times.

These time-dependent structural changes are not accounted for in static crystal graph representations and therefore cannot be directly predicted for the degradation of catalysts over thousands of hours of operation. The limitations are partially overcome by recent advances in machine-learning interatomic potentials, temporal graph neural networks, and physics-informed neural networks, which explicitly incorporate atomic trajectories and time-dependent structural evolution as graphs.

### 4.3 Mechanistic insight and descriptor discovery

In addition to prediction, machine learning provides powerful tools for interrogating the fundamental mechanisms driving catalytic activity, highlighting hidden correlations, identifying non-intuitive descriptors, and suggesting material designs that break through conventional limits.

#### 4.3.1 Uncovering hidden correlations and non-intuitive descriptors

Established descriptors like d-band centre, coordination number, and adsorption energies have been used to rationalise activity trends in traditional catalysis research. Although there are challenges associated with data mining, machine learning can discover new descriptors that capture previously unrecognized factors controlling performance (Mou L. et al., 2023; Mou T. et al., 2023). Feature importance analysis of trained models has shown which combinations of structural and electronic properties correlate best with catalytic outcomes, often identifying sets of features that describe catalytic activities more strongly than traditional single descriptors.

For high-entropy alloys, machine learning (ML) models trained on large-scale density functional theory datasets have identified d-band filling and differences in local electronegativity that enable mapping of adsorption energies to numerous active sites (Cao et al., 2025). While traditional heuristic approaches would leave these subtle correlations buried beneath more complex interactions, such composite descriptors may reveal insights into the true multi-element synergy that governs reactivity in multi-metallic systems. However, this is still impossible for catalysts with long-range order structures due to various degrees of disorder in coordination around active sites; it is well documented that structure–property correlations fail (Wang C. et al., 2025), yet ML models trained on local coordination environments discover structural motifs that correlate with activity and can thus enable rational design despite structural disorder.

#### 4.3.2 Informing the design of materials to break scaling relations

The scaling relations that limit conventional catalyst design linear correlations between the adsorption energies of different intermediates give rise to theoretical minimum overpotentials that cannot be overcome simply by surface engineering. Machine learning provides rules to identify materials for which these scaling relationships do not hold, allowing separate optimization of multiple reaction intermediaries (Govindarajan et al., 2019). Again, high-entropy alloys prove to be exemplary: their rich local coordination environments generate a distribution of adsorption sites suitable for the simultaneous optimisation of the binding of distinct intermediates. Conventional scaling between OH, O, and OOH adsorption energies breaks down for candidates identified from thousands of alloy compositions by ML models trained to predict these properties (Xu S. et al., 2024). Atomically dispersed catalysts tethered on purpose-built supports can reach nonconventional anchoring geometries via metal-support interactions that extend adsorbate bonding beyond the capabilities of three-dimensional macroscopic surfaces.

**FIGURE 11:**
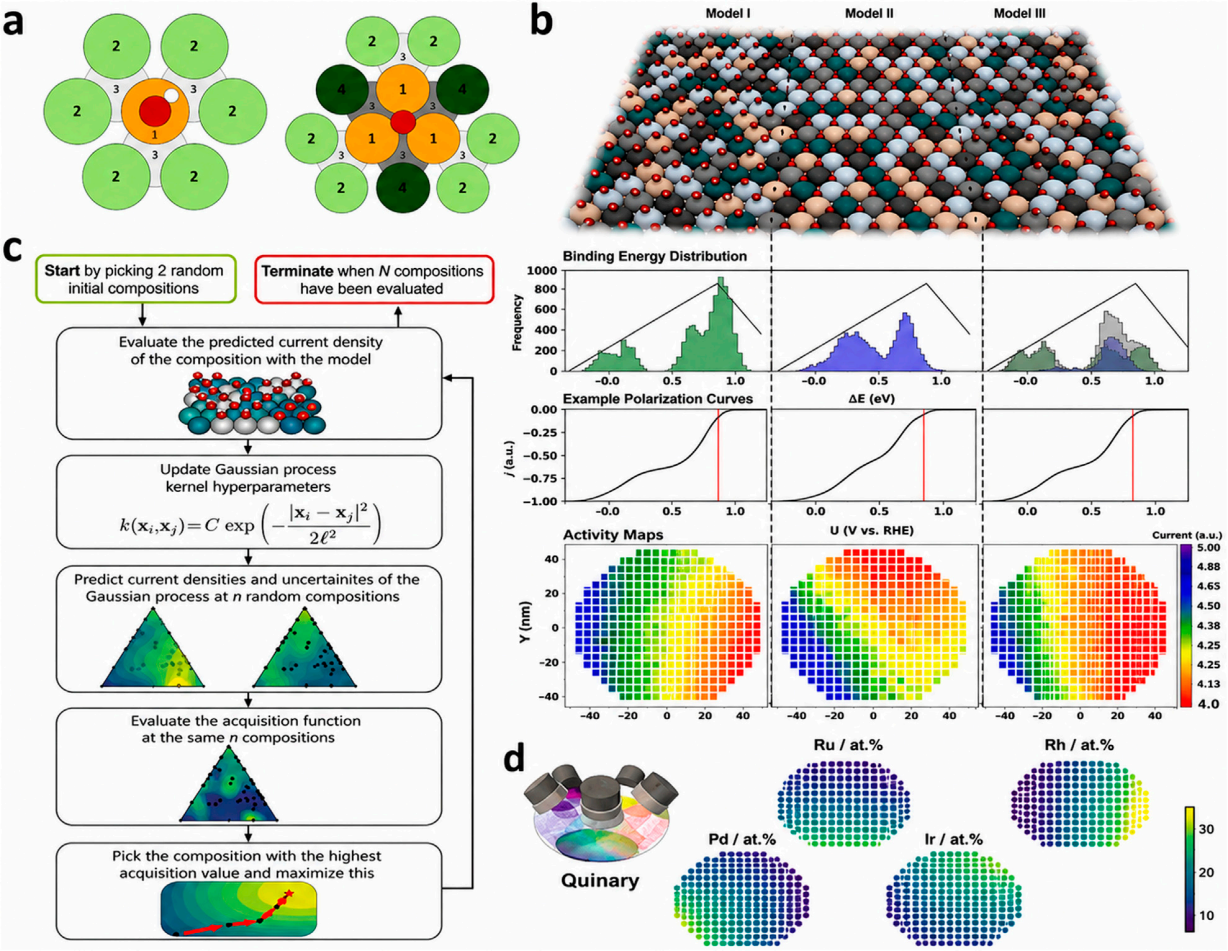
Machine learning-guided compositional optimization and activity mapping of multicomponent electrocatalyst systems. **(a)** Local atomic coordination environments used to parameterize adsorption-site configurations and nearest-neighbor interactions in complex alloy catalysts. **(b)** Surface-binding energy distributions, polarization behavior, and activity maps generated for compositionally complex catalytic surfaces using machine learning-assisted screening. **(c)** Bayesian optimization workflow for iterative exploration of multicomponent compositional spaces through uncertainty-guided acquisition and activity prediction. **(d)** High-throughput compositional mapping of quinary alloy catalyst libraries illustrating spatial distributions of elemental concentrations across co-sputtered electrocatalyst systems (Ding R. et al., 2024).

Figure 11 illustrates the use of accelerated exploration and optimization of complex multicomponent electrocatalyst systems by machine learning and Bayesian optimization strategies. The local coordination environments and the adsorption site configurations in compositionally complex alloys that control catalytic activity are parameterised as shown in (a). Subfigure (b) shows machine learning-based analysis of adsorption-energy distributions as well as electrochemical polarisation behaviour and spatial activity maps of the heterogeneous catalyst surfaces, which were used to identify high-performance compositional domains. Subfigure (c) illustrates the process of the Bayesian optimization workflow that dynamically balances exploration and exploitation to efficiently traverse the high-dimensional compositional space in the least number of experiments. Finally, as shown in subfigure (d), subfigure compositional mapping of quinary alloy libraries made by co-sputtering methods is performed to see how the automated experimentation and data-driven optimization are integrated in the current electrocatalyst discovery. This figure highlights the impact of machine learning on the rapid rational formulation and screening of complex alloy electrocatalysts, in sharp contrast to conventional trial-and-error approaches.

A conceptual diagram of the ΔG_OH vs. ΔG_OOH relationship, which shows the decoupling between the conventional linear correlation using ML guided strategies (high entropy alloys, single atom catalysts and support effects). The identification of materials with a broad adsorption energy distribution enables the design of catalysts with OER overpotentials below the theoretical volcano limit, a major finding of the mechanistic insight subsection.

### 4.4 Synthesis pathway optimization

To advance the proof-of-concept synthesis to experimental production, machine learning is being used to identify correlations between processing parameters and material properties, thereby informing synthesis optimisation. The morphology, particle size distribution, and defect density of the catalysts strongly depend on the synthesis conditions (temperature, pressure, precursor concentration, heating rate, atmosphere), and consequently on the catalyst performance. Machine learning models trained over synthetic–property datasets can learn such complicated relationships, thereby aiding in rational synthetic protocol optimization (Slautin et al., 2024). ML models can also be used to forecast how changes in synthesis parameters affect the size distribution, shape, and exposed facets of nanoparticle catalysts, all of which influence the availability of active sites and catalyst selectivity. Relationships between annealing conditions and vacancy concentration and type help to design materials where oxygen vacancies or metal cation defects act as active sites for OER or HER, for defect-engineered catalysts (Li et al., 2023). Bayesian optimisation then explores the high-dimensional synthesis space efficiently, and the optimal conditions are determined after a much smaller number of experiments than an experimentalist typically performs to achieve an optimal condition.

**FIGURE 12:**
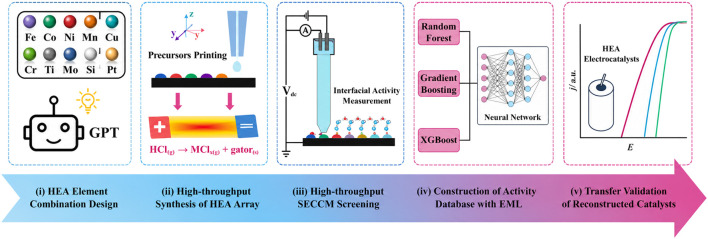
Machine learning-assisted high-throughput workflow for accelerated discovery and validation of high-entropy alloy electrocatalysts (i) Selection and optimization of high-entropy alloy elemental compositions using data-driven and machine learning-guided design strategies. (ii) High-throughput synthesis of compositionally diverse high-entropy alloy catalyst libraries through automated precursor printing approaches. (iii) Rapid electrochemical screening of intrinsic catalytic activity using scanning electrochemical cell microscopy (SECCM). (iv) Construction of electrocatalytic activity databases using ensemble machine learning models, including random forest, gradient boosting, XGBoost, and neural network frameworks. (v) Experimental transfer validation of machine learning-recommended high-performance electrocatalyst candidates (Jin et al., 2025).

The integrated machine learning workflow shown in Figure 12 can be used to narrow down the search, screen, and validate high-entropy alloy (HEA) electrocatalysts, thereby fast-tracking the electrocatalyst discovery process. Data-driven compositional design of multicomponent alloy systems is followed by automated high-throughput synthesis of catalyst arrays through precursor-printing strategies. Finally, scanning electrochemical cell microscopy is used to perform rapid intrinsic activity screening, efficiently obtaining a large electrochemical data set. These experimental results are then fed into ensemble machine learning models such as random forests, gradient boosting, XGBoost, and neural networks to accurately predict activity across a set of activity databases, thereby providing optimal catalyst compositions. Lastly, machine learning-recommended electrocatalysts are benchmarked against experiments using validated transfer learning methods. Overall, the figure illustrates how the combination of automation, multi-scale experimentation and machine learning significantly reduces catalyst optimization and discovery times for complex alloy systems. An AI-assisted optimization loop linking synthesis parameters (temperature, precursor ratio, heating rate) to catalyst morphology (particle size, defect density) and ultimately to electrocatalytic performance. The figure shows how Bayesian optimization and active learning navigate high-dimensional synthesis spaces to find optimal protocols with far fewer experiments, bridging the computational-experimental divide.

#### 4.4.1 Guiding the synthesis of predicted materials

The distance between computationally predicted candidates and experimentally obtainable materials is often quite large. Massively parallel screening of high-throughput synthesis outcomes from the literature with ML models can predict whether a composition and structure can be synthesized using conditions within reach (Szymanski et al., 2023). These predictors of synthesizability enable filtering of results from computational screening, allowing prioritisation of candidates likely to be amenable to experimental access. Autonomous synthesis platforms now close the loop: robotic systems execute ML-recommended synthesis protocols and characterize products, relaying results back to continuously refine models (Zhu et al., 2023). These self-driving laboratories run continuously, exploring synthesis spaces and optimizing protocols at orders of magnitude faster than human-driven experimentation. Such platforms have identified optimum synthesized conditions for newly predicted high-entropy alloy compositions, driving the computational catalyst predictions to catalysis.

### 4.5 Inverse design

The pinnacle of artificial intelligence in materials discovery is inverse design: defining target performance metrics and generating candidate material structures *de novo*, without forward screening of the existing candidates.

#### 4.5.1 Generative models for *de novo* catalyst design from target properties

Generative machine learning models (like variational autoencoders, generative adversarial networks, and diffusion models) learn the underlying distribution of stable crystal structures from high-throughput datasets of known materials. Once trained, these models can then create new structures conditioned on target properties: generate a crystal structure with a target adsorption energy, band gap, or formation energy (Zhou et al., 2026). For electrocatalyst design, generative models conditioned on target ΔGH values can suggest new compositions and structures predicted to exhibit optimal hydrogen binding. These candidates are often produced that strays significantly from known families of materials, probing chemical spaces that traditional intuition would never venture into. Property predictors then assess the generated candidates, and the most promising of these proceed to downstream validation via DFT calculations, synthesis, and testing. High-entropy alloys offer a particularly rich target of generative design: the enormous compositional space cannot be exhaustively mined, yet generative models can suggest multi-metallic compositions that are expected to yield optimum combinations of adsorption energies for multiple intermediates (Wang C. et al., 2025). Combined with synthesizability predictors and automated synthesis platforms, generative models hold the potential to achieve the long-desired goal of rational materials design in the truest sense, beginning with performance specifications and concluding with experimentally validated catalysts that meet them. This framework, which capitalizes on the synergy between activity prediction, stability forecasting, mechanistic insight, synthesis optimization, and inverse design, will define the new paradigm of ML-accelerated discovery of electrocatalysts. By inter-looping computation and experiment, prediction and validation, this approach holds the potential to accelerate the discovery of sustainable energy technologies by orders of magnitude.

## 5 The closed loop: integrating ML with computation and experiment

However, the most powerful impacts of machine learning in electrocatalysis arise not from isolated predictions but from synergistic combinations of ML and computational simulations, along with experimental validation. This iterative cycle of designing optimal experiments for discovery, followed by deploying improved mechanistic understanding to continuously feed predictive models with minimal human cognition required, creates orders-of-magnitude circumventing intermediating models from psychophysical predictions after reconciliation, through an accelerated hypothesis generation workflow, and in alignment (Tom et al., 2024).

### 5.1 The AI-guided workflow: prediction → validation → learning

Three interconnected stages of a closed-loop workflow. First, machine learning models are trained on historical data, and predictions are made across large chemical spaces to identify candidate materials that meet performance objectives. Properties. This enables high-velocity, iterative experimental campaigns based on these predictions that focus synthesis and characterisation efforts on the most promising candidates, rather than on random or heuristic searches. And finally, test results whether they are in accord (or variance) with predictions–become part of model training so that predictive accuracy can be refined incrementally and iteratively, and the domain of trustworthy inference expanded (Wang W. et al., 2023). This iterative fine-tuning proves very useful when dealing with complex and high-dimensional design spaces. Early-stage models using sparse data may exhibit high uncertainty or consistent bias. Each iteration provides a new set of training samples, labelled to address any gaps the model may have at that point, and active learning-based algorithms select which experiments to run to maximise their potential to improve the model’s performance. Over successive cycles, the combined approach converged on superior materials much more rapidly than is possible alone using computation or experimentation (Rodríguez-Martínez et al., 2021). The integration of Density Functional Theory calculations is an added workflow to this method. An alternative approach can be taken to train ML models on large DFT datasets, allowing them to learn how much quantum-chemical accuracy they achieve with minimal computational cost (Chen et al., 2025), rather than relying solely on costly, long-running methods based on experimental data. The ML surrogates process millions of candidates but take only the most promising on to experimental confirmation. This synergy of DFT-grounded ML predictions and experiments can be integrated into a multi-fidelity optimization loop, which can then use speed with precision.

### 5.2 Physics-informed neural networks: embedding domain knowledge

As good as purely data-driven neural networks are at prediction, they also tend to violate elementary physical laws, such as energy conservation, thermodynamic consistency, and rotational invariance, leading to predictions that are numerically inaccurate and physically impossible. A physics-informed method using neural networks bypasses this limitation by directly embedding knowledge of the governing equation into either the model architecture or the loss function (Wang S.-H. et al., 2021). The Governing equations (Schrödinger equation, Poisson–Boltzmann relations, and reaction–diffusion kinetics) are embedded as soft constraints during PINN training for electrocatalysis applications. For instance, a PINN trained to predict adsorption energies may minimize error with respect to DFT data while simultaneously satisfying consistency conditions: e.g., that the energy of a system should be invariant under rotations, or that adsorption energies for related intermediates obey physically motivated scaling relations (Omranpour et al., 2025). This physics-based rationale leads to fewer data requirements, enhanced extrapolation to out-of-distribution candidates, and ensures that the resulting predictions are physically meaningful at least for those areas of chemical space that are underrepresented in the training set. In a study utilizing electrochemical thermodynamics as background knowledge, the PINN method produces more accurate dissolution potentials and Pourbaix boundaries than purely data-driven methods for predicting electrocatalyst stability (Zhang H. et al., 2024). Incorporating the Nernst equation and transition-state theory as loss functions, these models will be governed by the fundamental relationships between potential, pH, and stability that drive the degradation of materials in catalysis. Despite learning solely experimental data from a small subset of each relevant environment, the resulting predictions remain robust over very different environments at the electrolyte level.

### 5.3 Active learning and Bayesian optimization for efficient exploration

The chemical space of potential electrocatalysts is effectively infinite, and exhaustive exploration can never become feasible, no matter how many computational resources one may throw at the problem. Both active learning and Bayesian optimization offer approaches to selectively search this space, allowing the model to choose which candidates are most informative (Deshmukh et al., 2024) for evaluation at each step. Active learning algorithms strike a balance between exploration of regions of chemical space where uncertainty in the model is high and exploitation, descent on predicted optimal material regions. This balance, learnt by itself using uncertainty quantification from ensemble methods, Bayesian neural networks, or Gaussian processes lead us to the right candidates where acquiring more data would best improve model robustness. For high-entropy alloy discovery, orders-of-magnitude savings in the total number of required DFT calculations have been achieved using active-learning workflows to identify stable and active compositions with minimal computational cost (Lan et al., 2023). Bayesian optimization generalizes this principle to continuous parameter spaces, synthesis conditions, composition ratios, processing variables, and expensive objective function evaluation (catalyst performance). A model (a probabilistic surrogate model, usually a Gaussian process) is constructed to represent the relationship between parameters and performance. An acquisition function is then used to choose the next set of parameters to evaluate, balancing between exploring uncertain areas and exploiting areas predicted to contain optima (Slautin et al., 2024). In the case of optimizing synthesis protocols, temperature, heating rate, and precursor concentration, Bayesian optimization finds optimal conditions in far fewer experiments than tried-and-true design-of-experiments approaches. Multi-objective Bayesian optimization generalizes this framework for problems with competing objectives: maximizing activity while minimizing cost, balancing HER and OER performance, and optimizing both activity and stability. Identifying a Pareto front illustrates the space of optimal trade-offs, which plays an important role in allowing researchers to select candidates that align with application-specific priorities (Xu S. et al., 2024).

### 5.4 Toward autonomous (self-driving) laboratories

The combination of machine learning, robotics, and high-throughput experimentation has now ushered in the era of fully autonomous discovery platforms consisting of self-driving laboratories that completely close the cycle between prediction and validation without human involvement (Tom et al., 2024).

Table 7 A comparative overview of five autonomous (self-driving) laboratory platforms: Robotic Chemist (Liverpool), CRESt (CMU/Toronto), AI-Robot Chemist (USTC), A-Lab (Berkeley), and Ada (Toronto). For each, it lists institution, key capabilities (robotic synthesis, multimodal characterization, closed-loop control), throughput (∼50–200 experiments/samples per day), ML integration level (Bayesian optimization, active learning, multimodal learning), and demonstrated application (OER catalysts, novel inorganic materials, etc.). This table directly supports the section’s narrative by showing how ML, robotics, and high-throughput experimentation are being integrated into fully autonomous discovery cycles.

**TABLE 7:** Comparison of autonomous (self-driving) laboratory platforms for electrocatalyst discovery.

Platform name	Institution	Key capabilities	Throughput	ML integration level	Demonstrated application	References
Robotic Chemist (Burger lab)	University of Liverpool	Mobile robot, liquid handling, synthesis, characterization	∼100 experiments/day	Bayesian optimization, active learning	Photocatalytic hydrogen production catalysts	Burger et al. (2020)
CRESt (Robotic platform)	Carnegie Mellon/University of Toronto	Multimodal (composition, microscopy, electrochemistry)	∼200 samples/day	Knowledge-assisted Bayesian optimization, multimodal learning	Multi-element OER catalysts (high-entropy oxides)	Zhang Z. et al. (2025)
AI-Robot Chemist (Martian catalyst)	University of Science and Technology of China	Robotic synthesis, in-situ characterization, closed-loop	∼50 experiments/day	Machine learning prediction + robotic execution	OER catalysts from Martian meteorites for ISRU	Zhu et al. (2023)
A-Lab (Berkeley Lab)	Lawrence Berkeley National Laboratory	Solid-state synthesis, powder X-ray diffraction	∼100 synthesis/characterization cycles/day	Natural language processing, active learning	Novel inorganic materials (structural predictions)	Szymanski et al. (2023)
Ada (Self-driving lab)	University of Toronto	Modular synthesis, automated testing, cloud-connected	Scalable (module-dependent)	Probabilistic programming, multi-fidelity optimization	Organic semiconductors, catalysts	Tom et al. (2024)

#### 5.4.1 Robotic synthesis and high-throughput electrochemical testing

**FIGURE 13:**
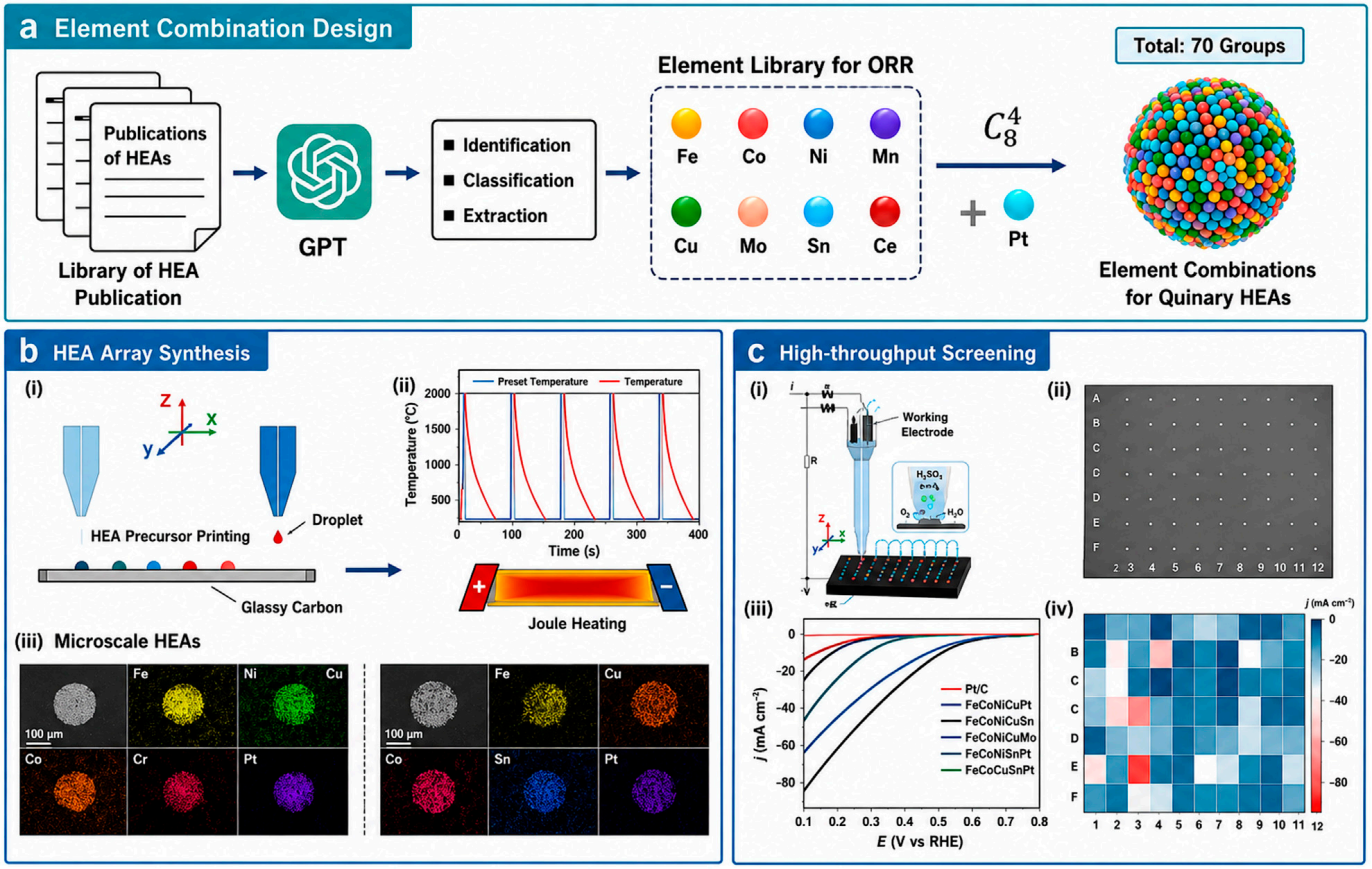
High-throughput synthesis and screening workflow for the discovery of high-entropy alloy electrocatalysts enabled using large language models. **(a)** Literature mining, elemental selection, classification and generation of quinary high-entropy alloy (HEA) compositions using the element-combination design framework with large language models. Precursor-printing approach for writing arrays of high-entropy alloys by automated micrometre-scale printing of precursors on conductive substrates. Rapid alloy synthesis and thermal processing using joule-heating temperature profile. **(a)** The image of synthesized microscale high-entropy alloy particles was obtained with scanning electron microscopy (SEM), and **(b)** the elemental mapping was performed using SEM. Electrochemical screening with high throughput **(c**-i**)** by scanning electrochemical cell microscopy (SECCM). Microscale alloy array electrochemical evaluation by optical imaging. **(c**-iii**)** Representative polarization curves showing that the electrocatalytic performance of the alloys varies. **(c**-iv**)** A relative activity heatmap of the best-designed high-entropy alloys for electrocatalytic reactions (Jin et al., 2025).

Figure 13 shows an integrated artificial intelligence-driven approach to high-throughput accelerated discovery and optimization of high-entropy alloy (HEA) electrocatalysts, where the large language models are used to search for relevant literature, automated synthetic protocol generation facilitates synthesis, and electrochemical screening provides the fundamental data to pinpoint the most suitable HEA electrocatalysts. Subfigure (a) illustrates the use of a large language model to extract both knowledge and objects from scientific literature, select candidates for a system, and produce compositional, different HEA systems for electrocatalytic applications. The automated microscale precursor-printing and rapid joule-heating synthesis method (subfigures b-i and b-ii) provide a fast and precise method to build an alloy library with customized thermal processing parameters, while ensuring scalable fabrication. Together with the scanning electron microscopy and the elemental mapping displayed on subfigure (b-iii), the resulting HEA particles are characterized, verifying the uniformity of the particles in terms of composition and formation of the particles’ microstructure at the microscale. In subfigures (c-i)–(c-iv), high-throughput electrochemical evaluation is performed by performing parallel screening, polarization measurements and activity heatmap; high-performance catalyst compositions are identified quickly. Together, these figures illustrate the dramatic power of combining automated materials synthesis, parallel electrochemical characterisation, and artificial intelligence to greatly improve the discovery and optimisation of catalysts within complex compositional spaces. High-throughput electrochemical testing; data incorporated into a high-throughput, autonomous discovery platform: ML prediction, robotic synthesis, automated characterisation (XRD, SEM, TEM), retraining ML. The figure is a representation of self-driving labs, and the whole design, build, test workflow is automated, further speeding up discovery by magnitudes.

Robotic platforms perform synthesis protocols with higher precision and reproducibility than humans can achieve. Liquid-handling robots dispense precursor solutions with microliter precision, automated high-throughput furnaces manage heating profiles with second-scale timing, and robotic arms shuffle samples between synthesis, characterization, and testing stations continuously (Zhu et al., 2023). These platforms discover electrocatalysts by synthesizing libraries of compositionally graded materials—thin-film composition spreads, nanoparticle arrays, and ink-jet printed electrode arrays that allow systematic probing of multinary systems. These libraries are screened in parallel via high-throughput electrochemical testing. Droplet cells, multichannel potentiators, and some optical screening methodologies measure activity, stability, and selectivity (Stevens et al., 2022) in the hundreds to thousands of samples daily. Automated pipelines for data analysis extract performance metrics, such as overpotential at specific current density, Tafel slopes, stability indices, and pipeline results directly back into ML retraining without any human data curation and also close the loop.

#### 5.4.2 Case studies of successful closed-loop discovery campaigns

Now the first closed-loop demonstrations of autonomous discovery in electrocatalysis have emerged, providing validation for the closed-loop paradigm (Burger et al., 2020) that detailed an autonomous system for discovering photocatalytic hydrogen production catalysts, achieving in days what would have taken months or years of human-directed experimentation. It used a mix of Bayesian optimization to select candidate compositions, robotic synthesis to create samples, and automated photoreactors to screen performance, going through iteration after iteration without human involvement. Other successful technologies have incorporated multimodal data chemical compositions, text embeddings from literature, microstructural images, and knowledge-assisted Bayesian optimization and robotic automation to discover multi-element catalysts for oxygen evolution catalysis (Zhang Z. et al., 2025) on the CRESt platform. Thus, the system was able to sample complex compositional spaces that were inaccessible for unimodal approaches and discard high-entropy oxide catalysts with activity above known materials. Importantly, the platform autonomously executed hundreds of synthesis, characterization, and testing cycles continuously over several weeks. Perhaps even more impressive was an AI-robot chemist that autonomously synthesized and optimized oxygen-producing catalysts from Martian meteorites (Zhu et al., 2023) This game-changing system, integrating ML-enabled prediction, robotic synthesis, and *in situ* characterization, screened thousands of potential catalyst formulations derived from Martian minerals and isolated the optimal compositions for catalysis relevant to oxygen evolution, potentially suitable for *in situ* resource utilization (ISRU) during Mars exploration. This work demonstrates the potential of closed-loop discovery: autonomous platforms that explore and optimize materials for important applications in scenarios where human intervention is infeasible due to resource limitations.

These successes foreshadow a future in which materials discovery becomes orders of magnitude faster. Instead of years-long cycles of experimentation by graduate students, closed-loop platforms running continuously can run thousands of discovery programs in parallel per year. Instead of sampling narrow compositional spaces around previously studied materials, such systems can traverse the entire chemical universe with only ML-guided predictions. Computation, ML, and robotics have all been making independent advances, but now they converge within a common paradigm that can catalyze a transition from the empirical art of electrocatalyst development to predictive processes facilitated by automation.

## 6 Critical challenges and research frontiers

Despite the tremendous advances presented in this review, leveraging machine learning to shape electrocatalyst design still faces daunting challenges that limit current capabilities and delineate future research trajectories. Overcoming these limitations data constraints, transferability deficiencies, and the interpretability versus predictive performance trade-off will ultimately determine whether ML achieves its transformative potential or lapses into a series of category demonstrations.

### 6.1 The data challenge: scarcity, bias, and the “small data” problem

The predictive power of any machine learning model is proportional to the amount, quality, and diversity of training data it consumes. This dependency creates an unavoidable bottleneck in electrocatalysis: data for developing transferable, accurate models of cathode processes is limited, expensive to generate, and consistently biased (Grajciar et al., 2018). Experimental data is costly to obtain, but the same is true for data generated using computational techniques: it exists in larger quantities than experimental data, though it also incurs high costs. The few advances that exist are still expensive: the tens of thousands of DFT calculations necessary to yield a high entropy alloy composition space (figure), it is still prohibitive even if resources are better trained (Tran et al., 2023): just one DFT calculation on complex oxide surface can require hours or years on supercomputers. Data from experiments are even more stringent. Due to the sensitivity of electrochemical measurements on electrode/electrolyte preparation as well as measurement protocols, reported overpotentials and Tafel slopes can have an orders-of-magnitude differences between labs, making dataset aggregation difficult. What the models must be learning to overcome is additional noise due to intrinsic variability in synthesis details, such as precursor purity, heating rates or aging time (Grim et al., 2020). These patterns of scarcity are exacerbated by systemic bias. The scientific literature is skewed toward positive results working catalysts, materials that have higher activity than relevant comparison compounds and successful syntheses. Nonsensical findings redundant representations, unvirtuous hybridizations, desperate syntheses have slowly slunk into the publication workflow well after they need to during both the training of balanced predictive models and prediction (Xu S. et al., 2024). This causes a positive bias in published datasets, leading to ML models that will err on the side of both overpredicting the probability of success for an unseen candidate and underpredicting the prevalence of failure modes.

To tackle these issues, we must band together at the community level. Open-access databases like the Materials Project, OQMD, or Open Catalyst provide computational data at unparalleled scales, but limited coverage of either oxide surfaces and/or amorphous materials and complex interfaces (Chanussot et al., 2021). Organizational Data Considerations of its Projects: Experimental data initiatives (i.e., the Dark Reactions Project) have included both successful and failed outcomes, yet widespread adoption of FAIR (Findable, Accessible, Interoperable, Reusable) data principles within organizational units has been slow off the mark. Automated extraction of data from the literature using natural language processing has a respite, but is still suboptimal: not only due to the heterogeneity of publication formats themselves, but also because of imprecision in the naming descriptors used to describe names, thereby threatening replicability (Suvarna et al., 2023). On shorter timescales, “small data” approaches (transfer learning, active learning, physics-informed regularization) will be critical. That is, you are pre-trained on high-dimensional computational datasets and then fine-tuned [on rare *in vivo* or CAR T-cell measurements, etc.,], thereby harnessing the best of both worlds. Active learning is a way to choose the experiments that will provide us with the most information, per measurement taken. Physics-informed models have leveraged domain expertise that helps inform where training examples are and are not, and in practice ensures predictions are physically plausible even when data is sparse.

### 6.2 The transferability gap: from idealized models to real catalysts and devices

Machine learning models that derive their training from idealized representations clean surfaces, perfect crystals, vacuum environments tend to underperform in predicting real catalyst performance under working conditions. The transferability gap has many causes, and each may require a different form of mitigation. Computational approaches typically only reproduce low-index surfaces of perfect crystals and ignore the steps, kinks, vacancies, and grain boundaries that dope catalyst faces in practice. These defects often are the actual active sites and behave in fundamentally different ways than ideal terrace sites (Resasco et al., 2022). Amorphous catalysts lack long-range order, making them a far cry from the structure-based representations that dominate innovative computational modeling of heterogeneous catalysis. Due to the numerous possible conformations, with only limited samples of defective and disordered structures (coming from computationally expensive *ab initio* molecular dynamics or approximated through more specialized sampling methods) it is currently possible to make some connection between these two worlds. Operational conditions introduce further complexity. Static conditions employed in DFT calculations are not able to account for the effects of electrolyte composition, pH, applied potential, and temperature on the structure and reactivity of catalysis. Recently included in Surface Pourbaix diagrams (Tables S12–S32) are observations suggesting that when considered under conditions of useful reaction, the stable surface termination is often different from its as-prepared or vacuum annealed form (Birdja et al., 2019) Reactivity is mediated by potential-dependent adsorption energies, solvation effects, and electrochemical double-layer structure, but all these are cheap to compute yet challenging to fold into ML training pipes.

These challenges are amplified by the chasm between lab testing and industrial execution. For the last 5 years, three-electrode lab cells in electrolysis have current densities (10–100 mA cm^−2^) 2 decades lower than 1–4 A cm^−2^ used in commercial electrolyzers. These phenomena result in the formation of a cluster with evolving bubbles, developing local pH gradients, and diffusion-controlled limitations, which creates an operational stage that is considerably less than idealized testing conditions (Zhang H. et al., 2024). Ionomer interactions, catalyst layer morphology, and water management start becoming limiting factors in measuring the performance of membrane electrode assemblies as compared to well-performing catalysts in rotating disk electrode measurements, leading to catastrophic failures. This gap will need to be filled, whether it’s through richer and more constrained device-level data (and multi-scale modelling that outputs bottom-line performance connected at the atomic level) or ML models trained on increasingly expansive datasets.

### 6.3 Interpretability vs. performance: moving beyond the “black box”

The most powerful machine learning models deep neural networks, graph convolutional networks, transformers are “black boxes,” opaque to humans in how they represent information internally and draw their conclusions. This is the section where that opacity creates tension; a very accurate model may be useful for predicting activity, but if we also cannot explain why such predictions are made, then not only does it lack contribution to fundamental knowledge but also rational design principles (Saidi, 2022). For example, in the catalysis community, we are not just interested in predictions, but want to develop design rules, controlling descriptors, and horizon-scan testable hypotheses about reaction mechanisms. Black-box models can be intractable with such goals their predictions do not even retain causal information that would help with subsequent finetuning (even if the training procedure was feasible). This means that even if a GNN predicts that a new alloy composition is “active,” the researcher only gets to know the prediction itself and no information on what electronic or geometric reason gave them this prediction. So this limitation endangers the construction of scientific knowledge and unsettles the argument for the model because of between-study heterogeneity in model suggestion.

Interpretable models linear regression, decision trees, symbolic regression sacrifice prediction for the sake of interpretability. Such functional forms will lend themselves relatively readily to interpretable features driving predictions and their signs, enabling the derivation of design rules (Cao et al., 2025) and judging mechanistic hypotheses. These types of models are extremely valuable for exploring new descriptors: symbolic regression can bootstrap analytic expressions that connect to catalytic activity by submitting physically relevant combinations of elemental properties in a way that adheres to trends over families of materials (Andersen et al., 2019). New efforts are attempting to straddle the divide, drawing on a blend of the best of both (Omranpour et al., 2025). Physics-informed neural networks embed domain knowledge into architectures such that learned representations for environments are encouraged to accord with physical intuition while preserving the expressivity of deep learning. Another direction is concept bottleneck models with interpretable intermediate representations adsorption energies, d-band centers, coordination numbers that ensure the prediction has to pass through human-interpretable structures while also threading that razor-thin needle of being more flexible than descriptor-based approaches, since ultimately they still boil down to end-to-end learning (Wang S.-H. et al., 2021).

The future, instead, will probably be in hybridization that shrewdly combines and recombines the advantages of various approaches. Such CNN zero false negatives if not all detections are selected out of huge compositional spaces by data-driven black-box high-capacity models. Stepwise interpretable models are then interrogated, and design rules/mechanisms are extracted that, in turn, can predict alternative avenues of study for deeper fundamental understanding. Active learning is an iteration between these modes limiting black box exploration using the insights learned from interpretable models, and utilizing black box predictions to push the boundaries of previously-known interpretable heuristics. It did not just accelerate discovery; it fundamentally reimagined how we understand and design catalytic materials.

**FIGURE 14:**
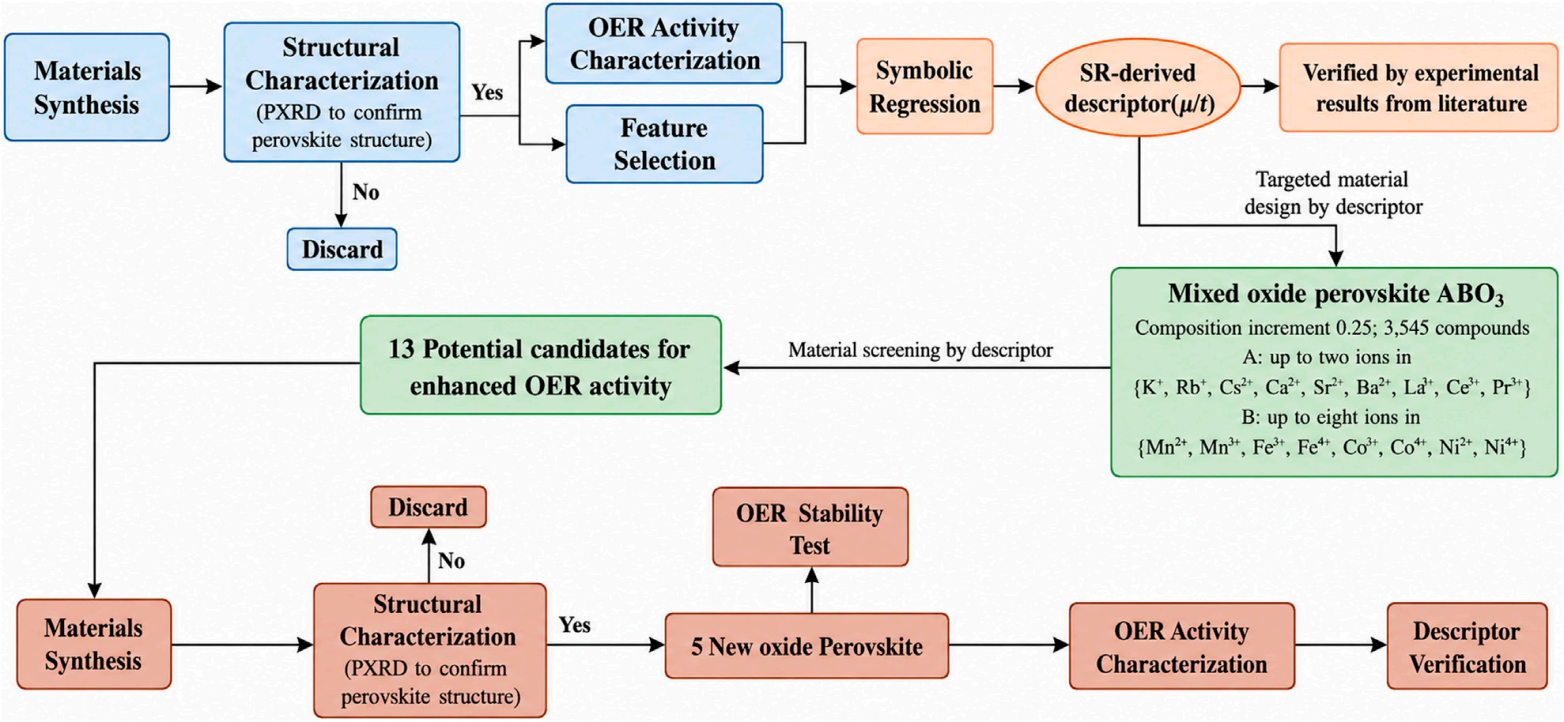
Symbolic regression-guided workflow for descriptor discovery and screening of perovskite electrocatalysts for oxygen evolution reaction (OER). The workflow illustrates the integration of materials synthesis, structural characterization, feature selection, symbolic regression, and experimental validation for accelerated discovery of high-performance mixed-oxide perovskite electrocatalysts. The upper pathway demonstrates dataset generation and descriptor extraction through oxygen evolution activity characterization and symbolic regression-based identification of structure–property relationships. The derived descriptor is subsequently applied for targeted screening of mixed-oxide ABO3 perovskite compositions across a broad compositional space. The lower pathway highlights experimental synthesis, structural verification, stability testing, and activity characterization of newly predicted perovskite candidates, followed by descriptor validation against experimentally measured OER performance (Hu et al., 2023).

Figure 14 presents a machine learning-assisted materials discovery framework employing symbolic regression for the identification of physically interpretable descriptors governing oxygen evolution reaction activity in mixed-oxide perovskite electrocatalysts. The workflow integrates experimental synthesis, structural characterization, feature engineering, and symbolic regression to establish quantitative structure–activity relationships capable of guiding catalyst design and screening. The derived descriptor is subsequently utilized to explore large compositional spaces of ABO_3_ perovskites and identify promising catalyst candidates with enhanced OER performance. Experimental validation through synthesis, electrochemical activity evaluation, and stability testing confirms the predictive capability of the descriptor-driven screening strategy. Overall, the figure highlights the growing importance of interpretable machine learning approaches in accelerating rational catalyst discovery while maintaining strong physical and chemical insight into electrocatalytic mechanisms. A visual summary of the three major challenges in ML-driven electrocatalysis: (i) data scarcity and bias (the “small data” problem), (ii) the transferability gap from idealised models to real devices, and (iii) the trade-off between model interpretability and predictive performance. The figure also lists emerging solutions (FAIR data, physics-informed models, active learning, self-driving labs), serving as a roadmap for future research.

## 7 The next-generation of AI in electrocatalysis

The direction of machine learning in electrocatalysis is toward increasingly sophisticated, integrated, and autonomous systems that will fundamentally change the way we discover, understand, and optimize catalytic materials. New paradigms generative artificial intelligence, foundation models, and multimodal learning have the potential to go beyond current capabilities, enabling functionality that would have been described as science fiction just a few years ago.

### 7.1 Generative AI and foundation models for materials science

The most foundational innovation on the horizon is what’s known as foundation models large-scale, pretrained neural networks that learn general representations from rich and diverse datasets, to be fine-tuned for specific downstream tasks (Chen et al., 2025). Foundation models with remarkable generative, reasoning, and zero-shot transfer capabilities like GPT and DALL-E have been pioneered in natural language processing and computer vision domains. Translating these architectures to the domain of materials science could have a similarly disruptive impact on catalyst discovery.

This means solving for the underlying physics to build an effective and generalizable model, because a materials foundation model trained on millions of crystal structures together with adsorption calculations and spectroscopic measurements would learn a representation rich in chemical and physical principles essentially capturing the “language” of materials within its millions of parameters (Zheng et al., 2025). Such models fine-tuned to specifically yield HER activity, stability predictions or synthetic pathways would require an order of magnitude fewer task-specific examples than a model trained from scratch, thereby directly addressing the challenge of data scarcity. Early implementations suggest that this approach has great promise: graph neural networks pretrained on the whole Materials Project database transfer well to diverse downstream tasks, with state-of-the-art performance achieved after minimal fine-tuning.

Generative models are more than prediction: they are creation. Variational autoencoders also learn continuous latent representations of crystal structures, which not only enables smooth interpolation between materials in the training data but can also generate new candidate crystals by sampling points from this learned manifold (Zhou et al., 2026). Conditional generation specifying properties of interest and generating structures that are likely to exhibit them works toward the inverse design paradigm: defining target performance features and suggesting candidate materials without extensive forward screening. This could mean producing alloy compositions with optimal and ΔGH values, oxide structures, and/or single-atom catalysts featuring tailored d-band centers or coordinated environments for electrocatalysis.

Diffusion models that learn to invert a gradual noising process have demonstrated state-of-the-art performance in generating high-fidelity and diverse samples across image and molecular domains. When used on crystal structures, these models could enable completely new materials under complex constraints thermodynamic stability, synthesizability, target adsorption energies and generate structural diversity across all of chemical space. Combined with property predictors and active learning, generative models have the potential to provide access to parts of chemical space that would be beyond any intuition from traditional approaches, thus dramatically expanding the boundaries of what can be discovered in catalysis.

### 7.2 Multimodal learning: fusing spectroscopy, microscopy, and performance data

**FIGURE 15:**
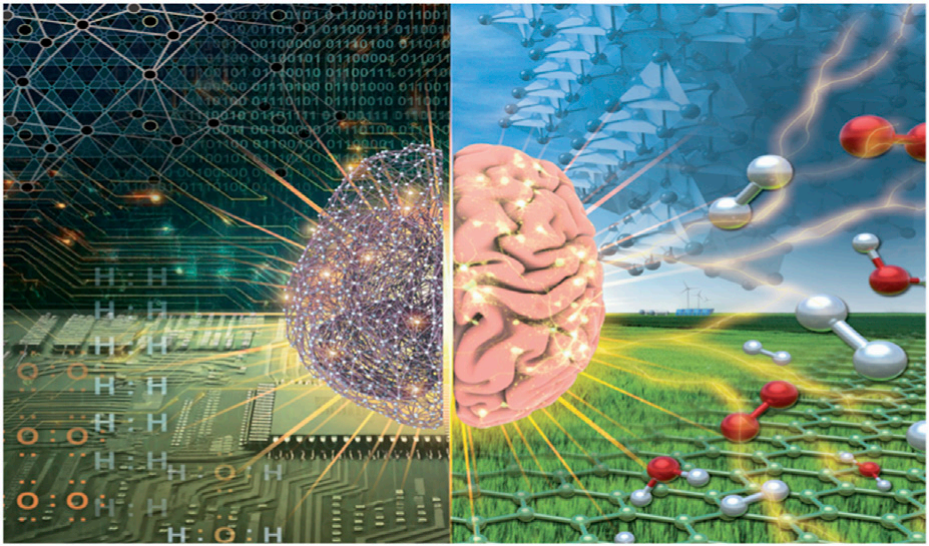
AI ecosystem for closed-loop autonomous electrocatalyst discovery. (Left/Virtual Domain): Multimodal fusion of text-mined literature databases, crystal connectivity graphs, and deep-learning interatomic potentials. (Right/Physical Domain): Direct translation of generative AI candidates into experimental testing—optimizing morphology, bonding, and gas evolution to turn empirical electrocatalysis into a predictive science (Ding R. et al., 2024).

Figure 15 conceptually illustrates the convergence of artificial intelligence, advanced materials science, and sustainable energy technologies in the future landscape of electrocatalyst discovery. The left half of the figure represents the computational and data-driven foundation of modern catalyst design, highlighting neural networks, digital infrastructures, large-scale data processing, and machine learning algorithms capable of learning complex physicochemical relationships. The central brain motif symbolizes the emergence of intelligent autonomous systems that integrate scientific reasoning, predictive modeling, and adaptive optimization. The right half of the figure transitions toward real-world energy applications, where atomistic materials engineering, molecular interactions, renewable energy integration, and catalytic reaction pathways are guided through AI-enabled discovery frameworks. Collectively, the figure emphasizes the transformative role of multimodal artificial intelligence, autonomous experimentation, and self-driving laboratories in accelerating the rational development of high-performance electrocatalysts for sustainable hydrogen production and future clean-energy technologies.

A two-domain AI ecosystem: the *virtual domain* fuses text-mined literature, crystal graphs, and deep-learning interatomic potentials; the *physical domain* translates generative-AI candidates into experimental testing (morphology, bonding, gas evolution). The figure illustrates the ultimate vision where multimodal data (spectra, microscopy, performance) and generative models work together to turn empirical electrocatalysis into a predictive, autonomous science.

Most applications of machine learning to date work in single data modalities crystal structures from databases, adsorption energies from DFT, and performance metrics from experiments. But human experts combine information across the different modalities: X-ray diffraction patterns indicate phase purity, electron microscopy images show particle size and morphology, X-ray photoelectron spectra indicate oxidation states and surface composition, and electrochemical measurements quantify activity and stability. Multimodal learning seeks to mirror and hasten this integrative reasoning through the melding of disparate data streams under common models (Tom et al., 2024).

From a technical standpoint, the challenge is about learning joint representations that match content from multiple sources. Early fusion involves concatenating features extracted across modalities before the final prediction; late fusion aggregates *post hoc* predictions from modality-specific models; while hybrid methods leverage cross-modal attention mechanisms that weight contributor information dynamically based on context. Multimodal models could predict the long-term stability of electrocatalysts from a combined analysis of initial electrochemical data and electron microscopy images, as well as X-ray spectra capturing degradation-relevant features that no single measurement reveals.

Multimodal learning also allows for potent inverse problems; given a transmission electron microscopy image of a synthesized catalyst, the trained model could output likely synthesis conditions, performance metrics, or stability in operation (Zhang Z. et al., 2025). This ability would shift characterization from responding to completion (which is retrospective) with high-throughput synthesis labor (not prospective guiding), to allowing researchers who use the same tool set as for experimental design up front, to interpret why this particular synthesis run worked or failed, and possible directions for correcting those conditions.

Then you can compound that even more with natural language, which will only widen the possibilities. While the scientific literature itself may be able to create a synthetic database of synthesized data, language models pretrained on this rich stream of details can fill in these synthesis recipes, characterization protocols, and performance claims across millions of publications that fill structured databases in unstructured text (Schrier et al., 2023). Multimodal models that synergize these text-extracted knowledge bases with experimental and computational data could answer queries currently requiring lengthy curation of the literature- “Which synthesis methods for NiFe layered double hydroxides have been reported, and how does their OER activity compare?”, What dopants are known to stabilize iridium oxide against dissolution in acid?” These models promise to accelerate research in that field by making the cumulative knowledge of that field machine-accessible and interpretable, far beyond what improved prediction alone would entail.

Generative AI, foundation models, and multimodal learning are converging to bring fundamentally new scales of materials discovery to the field. Instead of years-long cycles of hypothesis, experiment, and analysis, closed-loop platforms that integrate these capabilities could yield hundreds of discovery iterations a year. Rather than sampling dense compositional neighborhoods around known materials, generative models could suggest candidates that fill the chemical universe. Rather than reconstituting principles already documented in the literature, multimodal models could cohesively integrate knowledge scattered across millions of publications, revealing relationships that no human reader would be likely to detect.

Achieving this vision will require ongoing investment in data infrastructure, model development, and automated experimentation. Foundation models will not follow the trajectory of a single lab, but rather require coordination across the community working together (think Large Hadron Collider or Human Genome Project) to bring compute resources for training these kinds of models, which go well beyond anything possible by any single lab. Multimodal datasets corresponding to synthesis, characterization, and performance should be curated with institutional, standardized metadata for open access availability. Automation that can be achieved by having human operators do ML-recommended tests based on their intuition will ultimately lead to a performance plateau because, unless an operator knows when they are doing it wrong, they will not change what they are doing and get worse over time.

Although many would find the task intimidating, it’s a worthwhile labour that pays off. If successful, it would transform electrocatalyst development from an empirical art driven by personal know-how and luck into a predictive science, in which materials can be rationally designed, autonomously validated and swiftly deployed. For a field whose progress fuels the viability of sustainable energy technologies, few investments will pay off this much.

Table 8 represents the synthesis of the five major obstacles currently limiting ML adoption in electrocatalysis: data scarcity/bias, transferability gap, interpretability vs. performance, synthesizability of predicted materials, and early-stage autonomous discovery. For each challenge, the table describes its impact (e.g., overprediction of success, poor industrial relevance, hindered mechanistic understanding) and outlines future solutions (FAIR data principles, physics-informed models, self-driving labs, foundation models). This table serves as a roadmap, summarizing the challenges discussed in Section 6 and pointing toward the emerging research frontiers covered in Section 7.

**TABLE 8:** Key challenges and future directions in ML for electrocatalysis. Source (Grajciar et al., 2018; Resasco et al., 2022; Suvarna et al., 2023; Tom et al., 2024).

Challenge	Description	Impact	Future directions/Solutions
Data Scarcity and Bias	Limited experimental data; literature skewed toward positive results	Overprediction of success, poor generalizability	FAIR data principles, NLP-based data extraction, transfer learning, active learning
Transferability Gap	ML models trained on idealized surfaces fail to predict real catalyst performance under operating conditions	Poor industrial relevance, device-level failures	Multi-scale modeling, operando data integration, defect-inclusive datasets
Interpretability vs. Performance	High-accuracy models (GNNs, deep networks) are black-box; interpretable models have lower predictive power	Hinders mechanistic understanding and design rule derivation	Physics-informed models, concept bottlenecks, hybrid (interpretable + black-box) workflows
Synthesizability	Predicted materials often difficult to synthesize experimentally	Slow translation from computation to lab	Synthesis-aware ML predictors, autonomous synthesis platforms, Bayesian optimization of synthesis parameters
Autonomous Discovery	Integration of ML, robotics, and high-throughput experimentation remains nascent	Limited closed-loop demonstration at scale	Self-driving laboratories, multimodal learning, foundation models for materials science

## 8 Emerging AI technologies for autonomous catalyst discovery

Recent advances in artificial intelligence show that the field of catalyst discovery is rapidly transforming from modelling to autonomous research platforms. The transferability of their representation is expected to be achieved through millions of crystal structures and atomistic simulations used to train the foundation models, which is likely to reduce the need for task-specific training datasets and the multitude of foundation models across different catalyst families. Large Language Models (LLMs) are starting to support literature mining, hypothesis generation, automated synthesis planning, experimental protocol optimization, and scientific knowledge integration. In conjunction with retrieval-augmented generation, LLMs can continually update catalyst knowledge bases with new literature. Another key technological breakthrough is autonomous laboratories that incorporate robotics, high throughput experimentation, machine learning and Bayesian optimization. These automated laboratories synthesize and characterize catalysts, update models, and validate them in an iterative fashion, without requiring constant human supervision. Digital twins will combine physics-based simulations with real-time feedback from experiments to continuously monitor, predict and optimise the performance of electrolysers as the catalyst ages.

A roadmap to autonomous catalyst discovery can be envisioned in three practical stages. In the near-term (2025–2028), efforts will continue to focus on building high-quality databases of catalysts, enhancing uncertainty quantification, and creating interpretable machine learning models. Over the medium term (2028–2032), active learning, foundation models, multimodal AI, and autonomous laboratories will play a major role in faster optimization of catalysts along with cost reduction in experiments. In the future (post-2032), fully integrated closed-loop platforms that integrate the various components of robotics, machine learning, digital twins, operando characterization, and high throughput synthesis are expected to make it possible for self-driving laboratories that can autonomously discover, validate, and optimize electrocatalysts with minimal human involvement.

## 9 Conclusion

Machine learning approaches have revolutionised electrocatalyst discovery, shifting it away from its trial-and-error origins toward a data-driven predictive modelling paradigm. In this review, we have highlighted the connection between electrochemical principles, algorithmic developments, and self-propelled discovery platforms from a unified evolutionary perspective. The challenge of producing sustainable hydrogen still requires electrocatalysts to possess high activity, durability, and low cost within narrow design parameters. In this context, machine learning can be used to efficiently screen materials in high-dimensional materials space at comparatively low computational cost, thereby enabling the prediction and exploration of materials space. In this context, machine learning enables efficient virtual screening in the high-dimensional materials space at a comparatively low computational cost. Requires curated data infrastructures and a physically meaningful representation of structure, and a balance between model explanatory power and predictive ability. These methods can now be applied to generate catalysts with properties beyond conventional scaling relations and heuristics, ranging from regression models through graph-based neural networks. Emerging examples of closed-loop experimental platforms that incorporate machine learning and automated synthesis and characterisation are beginning to shorten design–build–test cycles, but data sparsity, bias, and discrepancies between model assumptions and the behaviour of operando catalysts under study persist. The realm of emerging generative and multimodal architectures holds various prospective paths towards inverse design and towards establishing spectroscopy–microscopy–electrochemical–mechanically coherent structure–property–performance relationships. Taken together, these are curbing towards a paradigm that is predictive, autonomous, and scalable, advancing the development of electrocatalysts towards the technological requirements of green hydrogen.

## References

[B1] Andersen M. Levchenko S. V. Scheffler M. Reuter K. (2019). Beyond scaling relations for the description of catalytic materials. ACS Catal. 9 (4), 2752–2759. 10.1021/acscatal.8b04478

[B2] Back S. Na J. Ulissi Z. W. (2021). Efficient discovery of active, selective, and stable catalysts for electrochemical H _2_ O _2_ synthesis through active motif screening. ACS Catal. 11 (5), 2483–2491. 10.1021/acscatal.0c05494

[B3] Batatia I. Kovács D. P. Simm G. N. C. Ortner C. Csányi G. (2023). MACE: higher order equivariant message passing neural networks for fast and accurate force fields. Available online at: http://arxiv.org/abs/2206.07697.

[B4] Birdja Y. Y. Pérez-Gallent E. Figueiredo M. C. Göttle A. J. Calle-Vallejo F. Koper M. T. M. (2019). Advances and challenges in understanding the electrocatalytic conversion of carbon dioxide to fuels. Nat. Energy 4 (9), 732–745. 10.1038/s41560-019-0450-y

[B5] Bowker M. DeBeer S. Dummer N. F. Hutchings G. J. Scheffler M. Schüth F. (2022). Advancing critical chemical processes for a sustainable future: challenges for industry and the Max planck–cardiff centre on the fundamentals of heterogeneous catalysis (FUNCAT). Angew. Chem. Int. Ed. 61 (50), e202209016. 10.1002/anie.202209016 PMC10099920 36351240

[B6] Burger B. Maffettone P. M. Gusev V. V. Aitchison C. M. Bai Y. Wang X. (2020). A mobile robotic chemist. Nature 583 (7815), 237–241. 10.1038/s41586-020-2442-2 32641813

[B7] Bushion J. Krishnan N. K. Kuo C. Thambusamy S. Govindasamy M. Eldesoky G. E. (2026). Delaminated two-dimensional Ti3C2Tx Mxene engineered gadolinium nitride nanostructures as an efficient bifunctional electrocatalyst for hydrogen and oxygen evolution reactions. J. Power Sources 688, 240521. 10.1016/j.jpowsour.2026.240521

[B8] Cao G. Yang S. Ren J.-C. Liu W. (2025). Electronic descriptors for designing high-entropy alloy electrocatalysts by leveraging local chemical environments. Nat. Commun. 16 (1), 1251. 10.1038/s41467-025-56421-9 39893203 PMC11787369

[B9] Chanussot L. Das A. Goyal S. Lavril T. Shuaibi M. Riviere M. (2021). Open catalyst 2020 (OC20) dataset and community challenges. ACS Catal. 11 (10), 6059–6072. 10.1021/acscatal.0c04525

[B10] Chen D. Shang C. Liu Z.-P. (2023). Machine-learning atomic simulation for heterogeneous catalysis. Npj Comput. Mater. 9 (1), 2. 10.1038/s41524-022-00959-5

[B11] Chen J. Huang X. Hua C. He Y. Schwaller P. (2025). A multi-modal transformer for predicting global minimum adsorption energy. Nat. Commun. 16 (1), 3232. 10.1038/s41467-025-58499-7 40185724 PMC11971357

[B12] Correa-Baena J.-P. Hippalgaonkar K. van Duren J. Jaffer S. Chandrasekhar V. R. Stevanovic V. (2018). Accelerating materials development via automation, machine learning, and high-performance computing. Joule 2 (8), 1410–1420. 10.1016/j.joule.2018.05.009

[B13] Deshmukh G. Wichrowski N. J. Evangelou N. Ghanekar P. G. Deshpande S. Kevrekidis I. G. (2024). Active learning of ternary alloy structures and energies. Npj Comput. Mater. 10 (1), 116. 10.1038/s41524-024-01256-z

[B14] Din M. A. U. Krishnan M. R. Alsharaeh E. H. (2025). Design strategies for cost-effective high-performance electrocatalysts in seawater electrolysis to produce hydrogen. J. Energy Chem. 102, 497–515. 10.1016/j.jechem.2024.10.047

[B15] Ding L. Li K. Wang W. Xie Z. Yu S. Yu H. (2024). Amorphous iridium oxide-integrated anode electrodes with ultrahigh material utilization for hydrogen production at industrial Current densities. Nano-Micro Lett. 16 (1), 203. 10.1007/s40820-024-01411-7 PMC11126398 38789605

[B16] Ding R. Chen J. Chen Y. Liu J. Bando Y. Wang X. (2024). Unlocking the potential: machine learning applications in electrocatalyst design for electrochemical hydrogen energy transformation. Chem. Soc. Rev. 53 (23), 11390–11461. 10.1039/D4CS00844H 39382108

[B17] Escayola S. Bahri-Laleh N. Poater A. (2024). % *V* _Bur_ index and steric maps: from predictive catalysis to machine learning. Chem. Soc. Rev. 53 (2), 853–882. 10.1039/D3CS00725A 38113051

[B18] Gao X. Chen Y. Wang Y. Zhao L. Zhao X. Du J. (2024). Next-generation green hydrogen: progress and perspective from electricity, catalyst to electrolyte in electrocatalytic water splitting. Nano-Micro Lett. 16 (1), 237. 10.1007/s40820-024-01424-2 PMC11226619 38967856

[B19] Gasteiger J. Giri S. Margraf J. T. Günnemann S. (2022). Fast and uncertainty-aware directional message passing for non-equilibrium molecules. Available online at: http://arxiv.org/abs/2011.14115.

[B20] Gasteiger J. Becker F. Günnemann S. (2024). GemNet: universal directional graph neural networks for molecules. Available online at: http://arxiv.org/abs/2106.08903.

[B21] Ghanekar P. G. Deshpande S. Greeley J. (2022). Adsorbate chemical environment-based machine learning framework for heterogeneous catalysis. Nat. Commun. 13 (1), 5788. 10.1038/s41467-022-33256-2 36184625 PMC9527237

[B22] Govindarajan N. Koper M. T. M. Meijer E. J. Calle-Vallejo F. (2019). Outlining the scaling-based and scaling-free optimization of electrocatalysts. ACS Catal. 9 (5), 4218–4225. 10.1021/acscatal.9b00532

[B23] Grajciar L. Heard C. J. Bondarenko A. A. Polynski M. V. Meeprasert J. Pidko E. A. (2018). Towards operando computational modeling in heterogeneous catalysis. Chem. Soc. Rev. 47 (22), 8307–8348. 10.1039/C8CS00398J 30204184 PMC6240816

[B24] Grim R. G. Huang Z. Guarnieri M. T. Ferrell J. R. Tao L. Schaidle J. A. (2020). Transforming the carbon economy: challenges and opportunities in the convergence of low-cost electricity and reductive CO_2_ utilization. Energy and Environ. Sci. 13 (2), 472–494. 10.1039/C9EE02410G

[B25] Guo J. Haghshenas Y. Jiao Y. Kumar P. Yakobson B. I. Roy A. (2024). Rational design of earth‐abundant catalysts toward sustainability. Adv. Mater. 36 (42), 2407102. 10.1002/adma.202407102 39081108

[B26] Hu Y. Chen J. Wei Z. He Q. Zhao Y. (2023). Recent advances and applications of machine learning in electrocatalysis. J. Mater. Inf. 3 (3). 10.20517/jmi.2023.23

[B27] Hu C. Hu Y. Zhang B. Zhang H. Bao X. Zhang J. (2024). Advanced catalyst design strategies and *in-situ* characterization techniques for enhancing electrocatalytic activity and stability of oxygen evolution reaction. Electrochem. Energy Rev. 7 (1), 19. 10.1007/s41918-024-00219-8

[B83] Jain A. Ong S. P. Hautier G. Chen W. Richards W. D. Dacek S. (2013). The Materials Project: A materials genome approach to accelerating materials innovation. APL Materials 1 (1), 011002. 10.1063/1.4812323

[B28] Jiang X. Chuah C. Y. Goh K. Wang R. (2021). A facile direct spray-coating of pebax® 1657: towards large-scale thin-film composite membranes for efficient CO2/N2 separation. J. Membr. Sci. 638, 119708. 10.1016/j.memsci.2021.119708

[B29] Jiang C. He H. Guo H. Zhang X. Han Q. Weng Y. (2024). Transfer learning guided discovery of efficient perovskite oxide for alkaline water oxidation. Nat. Commun. 15 (1), 6301. 10.1038/s41467-024-50605-5 39060252 PMC11282268

[B30] Jin Z. Gu D. Li P. Ye G. Zhu H. Wei K. (2025). Artificial intelligence-driven catalyst design for electrocatalytic hydrogen production: paradigm innovation and challenges in material discovery. Sustain. Chem. Energy Mater. 2, 100010. 10.1016/j.scenem.2025.100010

[B31] Keith J. A. Vassilev-Galindo V. Cheng B. Chmiela S. Gastegger M. Müller K.-R. (2021). Combining machine learning and computational chemistry for predictive insights into chemical systems. Chem. Rev. 121 (16), 9816–9872. 10.1021/acs.chemrev.1c00107 34232033 PMC8391798

[B32] Kibsgaard J. Chorkendorff I. (2019). Considerations for the scaling-up of water splitting catalysts. Nat. Energy 4 (6), 430–433. 10.1038/s41560-019-0407-1

[B33] Kolluru A. Shuaibi M. Palizhati A. Shoghi N. Das A. Wood B. (2022). Open challenges in developing generalizable large-scale machine-learning models for catalyst discovery. ACS Catal. 12 (14), 8572–8581. 10.1021/acscatal.2c02291

[B34] Lan J. Palizhati A. Shuaibi M. Wood B. M. Wander B. Das A. (2023). AdsorbML: a leap in efficiency for adsorption energy calculations using generalizable machine learning potentials. Npj Comput. Mater. 9 (1), 172. 10.1038/s41524-023-01121-5

[B35] Li J. (2022). Oxygen evolution reaction in energy conversion and storage: design strategies under and beyond the energy scaling relationship. Nano-Micro Lett. 14 (1), 112. 10.1007/s40820-022-00857-x PMC9051012 35482112

[B36] Li J. Wu N. Zhang J. Wu H.-H. Pan K. Wang Y. (2023). Machine learning-assisted low-dimensional electrocatalysts design for hydrogen evolution reaction. Nano-Micro Lett. 15 (1), 227. 10.1007/s40820-023-01192-5 37831203 PMC10575847

[B37] Liu H. Wang Y. Fan W. Liu X. Li Y. Jain S. (2023). Trustworthy AI: a computational perspective. ACM Trans. Intelligent Syst. Technol. 14 (1), 1–59. 10.1145/3546872

[B38] Luo Y. Zhang Z. Chhowalla M. Liu B. (2022). Recent advances in design of electrocatalysts for high‐current‐density water splitting. Adv. Mater. 34 (16), 2108133. 10.1002/adma.202108133 34862818

[B39] Motagamwala A. H. Ball M. R. Dumesic J. A. (2018). Microkinetic analysis and scaling relations for catalyst design. Annu. Rev. Chem. Biomol. Eng. 9 (1), 413–450. 10.1146/annurev-chembioeng-060817-084103 29641915

[B40] Mou L. Han T. Smith P. E. S. Sharman E. Jiang J. (2023). Machine learning descriptors for data‐driven catalysis Study. Adv. Sci. 10 (22), 2301020. 10.1002/advs.202301020 PMC10401178 37191279

[B41] Mou T. Pillai H. S. Wang S. Wan M. Han X. Schweitzer N. M. (2023). Bridging the complexity gap in computational heterogeneous catalysis with machine learning. Nat. Catal. 6 (2), 122–136. 10.1038/s41929-023-00911-w

[B42] Musaelian A. Batzner S. Johansson A. Sun L. Owen C. J. Kornbluth M. (2022). Learning local equivariant representations for large-scale atomistic dynamics. Available online at: http://arxiv.org/abs/2204.05249. 10.1038/s41467-023-36329-y PMC9898554 36737620

[B43] Oh S.-H. V. Yoo S.-H. Jang W. (2024). Small dataset machine-learning approach for efficient design space exploration: engineering ZnTe-based high-entropy alloys for water splitting. Npj Comput. Mater. 10 (1), 166. 10.1038/s41524-024-01341-3

[B44] Omranpour A. Elsner J. Lausch K. N. Behler J. (2025). Machine learning potentials for heterogeneous catalysis. ACS Catal. 15 (3), 1616–1634. 10.1021/acscatal.4c06717

[B45] Peng J. Schwalbe-Koda D. Akkiraju K. Xie T. Giordano L. Yu Y. (2022). Human- and machine-centred designs of molecules and materials for sustainability and decarbonization. Nat. Rev. Mater. 7 (12), 991–1009. 10.1038/s41578-022-00466-5

[B46] Pham C. V. Escalera‐López D. Mayrhofer K. Cherevko S. Thiele S. (2021). Essentials of high performance water electrolyzers – from catalyst layer materials to electrode engineering. Adv. Energy Mater. 11 (44), 2101998. 10.1002/aenm.202101998

[B47] Qian X. Yoon B.-J. Arróyave R. Qian X. Dougherty E. R. (2023). Knowledge-driven learning, optimization, and experimental design under uncertainty for materials discovery. Patterns 4 (11), 100863. 10.1016/j.patter.2023.100863 38035192 PMC10682757

[B48] Qian F. Cao D. Chen S. Yuan Y. Chen K. Chimtali P. J. (2025). High-entropy RuO2 catalyst with dual-site oxide path for durable acidic oxygen evolution reaction. Nat. Commun. 16 (1), 6894. 10.1038/s41467-025-61763-5 40715041 PMC12297328

[B49] Quaino P. Juarez F. Santos E. Schmickler W. (2014). Volcano plots in hydrogen electrocatalysis – uses and abuses. Beilstein J. Nanotechnol. 5, 846–854. 10.3762/bjnano.5.96 24991521 PMC4077405

[B50] Reiser P. Neubert M. Eberhard A. Torresi L. Zhou C. Shao C. (2022). Graph neural networks for materials science and chemistry. Commun. Mater. 3 (1), 93. 10.1038/s43246-022-00315-6 36468086 PMC9702700

[B51] Resasco J. Abild-Pedersen F. Hahn C. Bao Z. Koper M. T. M. Jaramillo T. F. (2022). Enhancing the connection between computation and experiments in electrocatalysis. Nat. Catal. 5 (5), 374–381. 10.1038/s41929-022-00789-0

[B52] Rodríguez-Martínez X. Pascual-San-José E. Campoy-Quiles M. (2021). Accelerating organic solar cell material’s discovery: high-throughput screening and big data. Energy and Environ. Sci. 14 (6), 3301–3322. 10.1039/D1EE00559F 34211582 PMC8209551

[B85] Rosen A. S. Iyer S. M. Ray D. Yao Z. Aspuru-Guzik A. Gagliard L. (2021). Machine learning the quantum-chemical properties of metal–organic frameworks for accelerated materials discovery. Matter 4 (5), 1578–1597. 10.1016/j.matt.2021.02.015 PMC8085598 33982029

[B53] Saidi W. A. (2022). Emergence of local scaling relations in adsorption energies on high-entropy alloys. Npj Comput. Mater. 8 (1), 86. 10.1038/s41524-022-00766-y

[B54] Salami R. Liu T. Han X. Zheng Y. (2025). Machine learning application in thermal CO2 hydrogenation: catalyst design, process optimization, and mechanism insights. Adv. Powder Mater. 4 (6), 100333. 10.1016/j.apmate.2025.100333

[B55] Schrier J. Norquist A. J. Buonassisi T. Brgoch J. (2023). In pursuit of the exceptional: research directions for machine learning in chemical and materials science. J. Am. Chem. Soc. 145 (40), 21699–21716. 10.1021/jacs.3c04783 37754929

[B56] Schütt K. T. Sauceda H. E. Kindermans P.-J. Tkatchenko A. Müller K.-R. (2018). SchNet – a deep learning architecture for molecules and materials. J. Chem. Phys. 148 (24), 241722. 10.1063/1.5019779 29960322

[B57] Sead F. F. Reddy M. S. Sahu B. N. Sundharam S. Kumar S. Pramanik A. (2026). Dual-function MOF@covalent triazine framework – au hybrid for visible-light-driven photocatalytic hydrogen evolution. J. Environ. Chem. Eng. 14 (3), 122992. 10.1016/j.jece.2026.122992

[B58] Seh Z. W. Kibsgaard J. Dickens C. F. Chorkendorff I. Nørskov J. K. Jaramillo T. F. (2017). Combining theory and experiment in electrocatalysis: insights into materials design. Science 355 (6321), eaad4998. 10.1126/science.aad4998 28082532

[B59] Slautin B. N. Liu Y. Funakubo H. Vasudevan R. K. Ziatdinov M. Kalinin S. V. (2024). Bayesian conavigation: dynamic designing of the material digital twins via active learning. ACS Nano 18 (36), 24898–24908. 10.1021/acsnano.4c05368 39183496

[B60] Sokolov M. Exner K. S. (2024). Is the *O vs. *OH scaling relation intercept more relevant than the *OOH vs. *OH intercept to capture trends in the oxygen evolution reaction? Chem. Catal. 4 (7), 101039. 10.1016/j.checat.2024.101039

[B61] Srinivasan S. K. Jayaraman S. Sekar B. K. Rajendran A. K. Jayabal R. Prabhakar P. (2026). Integrated monitoring and lifecycle assessment of green hydrogen, ammonia, and synthetic fuels: advancing environmental sustainability and carbon traceability in the clean energy transition. Environ. Prog. and Sustain. Energy 45 (2), e70328. 10.1002/ep.70328

[B84] Stevanović V. Lany S. Zhang X. Zunger A. (2012). Correcting density functional theory for accurate predictions of compound enthalpies of formation: Fitted elemental-phase reference energies. Physical Review 85, 11510. 10.1103/PhysRevB.85.115104

[B62] Stevens M. B. Anand M. Kreider M. E. Price E. K. Zeledón J. Z. Wang L. (2022). New challenges in oxygen reduction catalysis: a consortium retrospective to inform future research. Energy and Environ. Sci. 15 (9), 3775–3794. 10.1039/D2EE01333A

[B63] Suvarna M. Vaucher A. C. Mitchell S. Laino T. Pérez-Ramírez J. (2023). Language models and protocol standardization guidelines for accelerating synthesis planning in heterogeneous catalysis. Nat. Commun. 14 (1), 7964. 10.1038/s41467-023-43836-5 38042926 PMC10693572

[B64] Szymanski N. J. Rendy B. Fei Y. Kumar R. E. He T. Milsted D. (2023). An autonomous laboratory for the accelerated synthesis of inorganic materials. Nature 624 (7990), 86–91. 10.1038/s41586-023-06734-w 38030721 PMC10700133

[B65] Tom G. Schmid S. P. Baird S. G. Cao Y. Darvish K. Hao H. (2024). Self-Driving laboratories for chemistry and materials science. Chem. Rev. 124 (16), 9633–9732. 10.1021/acs.chemrev.4c00055 39137296 PMC11363023

[B66] Tong Y. Wang L. Hou F. Dou S. X. Liang J. (2022). Electrocatalytic oxygen reduction to produce hydrogen peroxide: rational design from single-atom catalysts to devices. Electrochem. Energy Rev. 5 (3), 7. 10.1007/s41918-022-00163-5 PMC9437407 37522152

[B67] Tran R. Lan J. Shuaibi M. Wood B. M. Goyal S. Das A. (2023). The open catalyst 2022 (OC22) dataset and challenges for oxide electrocatalysts. ACS Catal. 13 (5), 3066–3084. 10.1021/acscatal.2c05426

[B68] Wang S. Lu A. Zhong C.-J. (2021). Hydrogen production from water electrolysis: role of catalysts. Nano Converg. 8 (1), 4. 10.1186/s40580-021-00254-x 33575919 PMC7878665

[B69] Wang S.-H. Pillai H. S. Wang S. Achenie L. E. K. Xin H. (2021). Infusing theory into deep learning for interpretable reactivity prediction. Nat. Commun. 12 (1), 5288. 10.1038/s41467-021-25639-8 34489441 PMC8421337

[B70] Wang T. Cao X. Jiao L. (2022). PEM water electrolysis for hydrogen production: fundamentals, advances, and prospects. Carbon Neutrality 1 (1), 21. 10.1007/s43979-022-00022-8

[B71] Wang W. Jiang X. Tian S. Liu P. Lookman T. Su Y. (2023). Alloy synthesis and processing by semi-supervised text mining. Npj Comput. Mater. 9 (1), 183. 10.1038/s41524-023-01138-w

[B72] Wang C. Wang B. Wang C. Li A. Chang Z. Wang R. (2025). A machine learning model with minimize feature parameters for multi-type hydrogen evolution catalyst prediction. Npj Comput. Mater. 11 (1), 111. 10.1038/s41524-025-01607-4

[B73] Wang J. Liu Y. Yang G. Jiao Y. Dong Y. Tian C. (2025). MXene-Assisted NiFe sulfides for high-performance anion exchange membrane seawater electrolysis. Nat. Commun. 16 (1), 1319. 10.1038/s41467-025-56639-7 39900925 PMC11790850

[B74] Wang X. Pi W. Hu S. Bao H. Yao N. Luo W. (2025). Boosting oxygen evolution reaction performance on NiFe-Based catalysts through d-Orbital hybridization. Nano-Micro Lett. 17 (1), 11. 10.1007/s40820-024-01528-9 PMC11427650 39325091

[B75] Wu L. Xu Y. Wang Q. Zou X. Pan Z. Leung M. K. H. (2025). Direct seawater electrolysis for green hydrogen production: electrode designs, cell configurations, and system integrations. Energy and Environ. Sci. 18 (10), 4596–4624. 10.1039/D5EE01093D

[B76] Xu S. Chen Z. Qin M. Cai B. Li W. Zhu R. (2024). Developing new electrocatalysts for oxygen evolution reaction via high throughput experiments and artificial intelligence. Npj Comput. Mater. 10 (1), 194. 10.1038/s41524-024-01386-4

[B77] Xu W. Diesen E. He T. Reuter K. Margraf J. T. (2024). Discovering high entropy alloy electrocatalysts in vast composition spaces with multiobjective optimization. J. Am. Chem. Soc. 146 (11), 7698–7707. 10.1021/jacs.3c14486 38466356 PMC10958507

[B78] Zhang H. Chen L. Dong F. Lu Z. Lv E. Dong X. (2024). Dynamic transformation of active sites in energy and environmental catalysis. Energy and Environ. Sci. 17 (18), 6435–6481. 10.1039/D4EE02365J

[B79] Zhang Z. Ren Z. Hsu C.-W. Chen W. Hong Z.-W. Lee C.-F. (2025). A multimodal robotic platform for multi-element electrocatalyst discovery. Nature 647 (8089), 390–396. 10.1038/s41586-025-09640-5 40987343

[B80] Zheng P. Han Z. Su B.-L. Xu G. (2025). Catal-GPT: AI-driven directed efficient design framework for catalysts. Natl. Sci. Rev. 12 (9), nwaf299. 10.1093/nsr/nwaf299 40937449 PMC12421571

[B81] Zhou D. Yang R. Jia Z. Cai Y. Zhao L. Guo L. (2026). A practical inverse design approach for high-entropy catalysts using generative AI. Nat. Synth. 5, 730–739. 10.1038/s44160-025-00983-5

[B82] Zhu Q. Huang Y. Zhou D. Zhao L. Guo L. Yang R. (2023). Automated synthesis of oxygen-producing catalysts from Martian meteorites by a robotic AI chemist. Nat. Synth. 3 (3), 319–328. 10.1038/s44160-023-00424-1

